# Scientific, societal and pedagogical approaches to tackle the impact of climate change on marine pollution

**DOI:** 10.1038/s41598-021-82421-y

**Published:** 2021-02-03

**Authors:** Tiago M. Alves, Eleni Kokinou, Marie Ekström, Andreas Nikolaidis, Georgios C. Georgiou, Anastasia Miliou

**Affiliations:** 1 3D Seismic Lab-School of Earth and Ocean Sciences, Cardiff University-Main Building, Park Place, Cardiff, CF10 3AT UK; 2 Laboratory of Applied Geology and Hydrogeology, Department of Agriculture, Hellenic Mediterranean University, P.O. Box 1939, 71004 Heraklion, Crete Greece; 3 Institute of Computer Science, Foundation for Research and Technology-Hellas, 70013 Heraklion, Crete Greece; 4 Oceanography Centre, University of Cyprus, P.O. Box 20537, 1678 Nicosia, Cyprus; 5 Archipelagos Institute of Marine Conservation, Pythagorio 83 103, P.O. Box 42, Samos, Greece

**Keywords:** Environmental impact, Psychology and behaviour, Physical oceanography

## Abstract

Marine pollution impacts coastal nations around the world, and more so: (a) in confined maritime areas with significant marine traffic, (b) where exploitation of natural and mineral resources is taking place, or (c) in regions witnessing pressure from tourism, local population growth, and industry. In this work, Digital Elevation Models, hydrographic, and climatic data are used together with computer simulations to understand the control of climate change on marine pollution. The results show that different climate change signals can potentially alter the flow and concentration of pollution in the European Seas, when compared to the present day. Ultimately, this work identifies the main sources of marine pollution as: (1) rivers and streams near cities and industrialised areas, (2) coastal areas experiencing sudden demographic pressures, (3) offshore shipping lanes in which oil and other marine debris are released, and (4) areas of rugged seafloor where industrial fishing takes place. This paper finishes by describing new educational material prepared to teach school children around the world. It explains why how a new training curriculum and e-game developed by Sea4All can be crucial in future Environmental Education and Education for a Sustainable Development.

## Introduction

Marine pollution varies in its relative importance to include pollutants such as agricultural fertilizers and chemical pesticides, litter discarded during large cultural events, debris and objects freely discarded in regions subject to mass tourism, plastic and domestic litter derived from large cities, oil and chemical spills around industrial compounds and, rather frequently, domestic and industrial waste disposed of in rivers^1,2^. These diverse types of pollutants can be grouped in two classes: (a) chemical or oil spills, and (b) releases of floating (and other) objects into the sea, a classification adopted throughout this work for the purposes of analyzing the main sources of marine pollution in the world's seas. Floating objects, comprising multiple types of debris, can ultimately sink onto the sea bottom and are subjected to similar current and wave action when released. Sea currents and waves will contribute to their disaggregation, or lead to their increase in density (due to water absorption) to facilitate the sinking of objects such as plastic, drifting wood or synthetic materials (e.g. microbeads)^2^.

Chemical spills and floating objects sourced from land are often the byproduct of substandard water treatment, inefficient litter collection procedures, or relate to an incomplete environmental monitoring of industrial and commercial pollutants^3,4^. The seasonal impact of tourism, and an increasing tendency for the industrialization of coastal areas around major shipping routes, compound this problem to create environmental concerns in numerous maritime countries^5,6^. Adding to deliberate or accidental pollution near waterways, large cities are known producers of litter, plastic and other types of floating objects^7^. A key example of the impact of tourism on urban areas is the recent effect of Covid-19 restrictions on Venice’s canals. Here, the absence of bottom and water winnowing by motor boats and other vessels (gondolas, rowing boats) during the first wave of the Covid-19 pandemic dramatically improved the transparency of waters around Venice, except in areas subject to fishing and commercial shipping^8^.

This work goes a step further by proposing that the relevance of rivers to marine pollution, relative to other sources of oil, chemical and floating objects, will vary in the future as the signature of climate change varies across Europe. Estimates of change based on simulations from an ensemble of regional climate models (RCM) suggest that rainfall is likely to increase in Northern Europe, whilst southern regions will become drier, especially in summer^9^. Estimates based on a smaller RCM ensemble used in combination with a hydrological model suggest that changes in water runoff echo those predicted for rainfall. They point to increased runoff in parts of Sweden, Norway, Northeast Europe, Austria, the northwest Balkans and Hungary, but a widespread and intense decrease in water runoff along the Iberian and Balkan coasts^10^. These projections of change agree with current trends concerning river floods, where increasing precipitation in Northwest Europe during autumn and winter have led to increased flooding. Conversely, decreasing precipitation and increasing evaporation in Southern Europe has had the opposite effect^11^. Authors also document decreasing floods in Eastern Europe, the key drivers being decreasing snow cover and snowmelt due to warmer temperatures. Therefore, understanding change to mean and high (or extreme) river flows is important to assessing the contribution of pollutants from land sources, and should be considered together with assessment of changes to tourism patterns, such as number of ships, recreational yachts and visitors to a particular region.

A specific aspect of parts of Southern Europe, namely the Mediterranean and Black Seas, is that their onshore topography is dominated by mountain ranges on which large rates of erosion, sediment transport and river water flow are recorded during seasonal flash-flood events^12,13^. These flash floods enhance the transport of sediment and water from the hinterland to coastal areas during early fall and winter, at the same time carrying floating objects and chemical pollutants to near-shore areas via the same rivers and streams where they were accidentally disposed of during the dry season^13^. Importantly, assessments of European flooding trends indicate that flood discharges are decreasing in Southern Europe^14^, whereas discharges in medium to large catchments are relatively high in a European context. They further suggest that floods in small catchments of Southern Europe will increase due to enhanced convective storms and land-use changes^14^.

**Figure 1:**
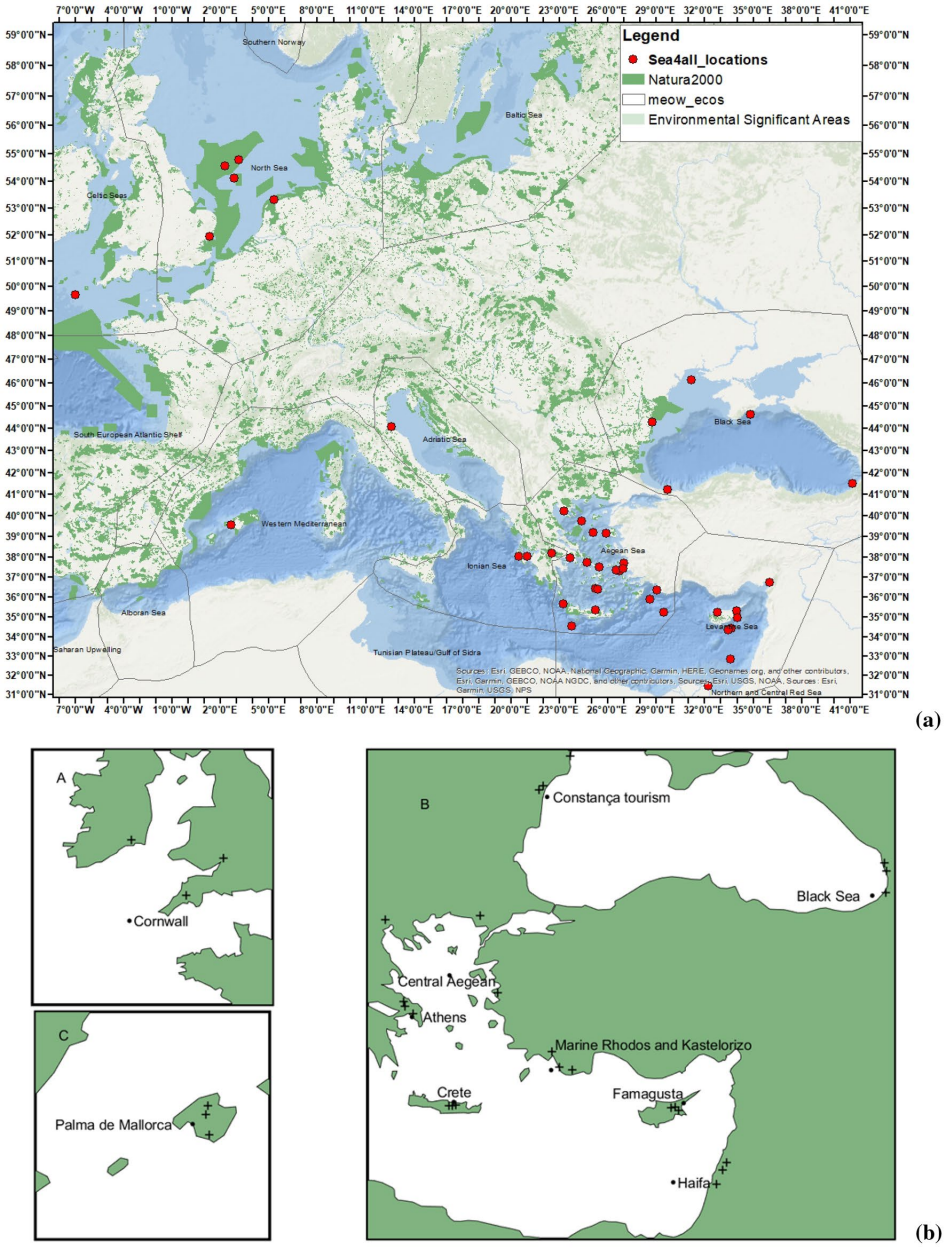
General maps of the study area. (**a**) Environmentally significant areas and Natura 2000 sites in Europe shown together with the locations of the 43 pollution scenarios implemented in the context of the Sea4All project (www.sea4all-project.eu). (**b**) Relative location of sites in which river flow data were gathered from the Service for Water Indicators in Climate Change Adaptation (SWICCA) database. Figure (**a**) was created using ArcGIS (https://pro.arcgis.com/en/pro-app/get-started/get-started.htm#:~:text=ArcGIS%20Pro%20is%20the%20latest,elements%20of%20the%20user%20interface and https://www.marathondata.gr/). Natura2000 data are from http://www.eea.europa.eu/data-and-maps/data/natura-2/natura-2000-spatial-data/.

This study focusses on the principal sources of pollution in Europe, and how future and seasonal climate will control their relative importance. Based on the results of the ERASMUS + Sea4All project (www.sea4all-project.eu), we demonstrate the importance of considering local physiographical characteristics in combination with regionally specific hydroclimate projections when assessing plausible trajectories of marine pollution over the coming decades. For the purposes of this work, 43 accident scenarios were modelled using meteorological and oceanographic data for specific seasonal conditions (Fig. 1). In a second stage, the accident scenarios and other data were used to promote a better pedagogical and societal awareness of the impacts of marine pollution. In a third stage, we created online education material, games and tools to promote the environmental awareness and consciousness in 10–14 year-old pupils, as a priority age group, and within the educational community (i.e., teachers and educators). This was the first time interacting scientific and pedagogical approaches were applied in pan-European context to educate school pupils around the world (https://www.sea4all-project.eu/edu/).

## Methods and datasets

Bathymetric/elevation, geomorphologic, meteorologic and oceanographic analyses, together with marine ecology parameters for the Mediterranean, Black Sea and Atlantic-UK waters, were fused in the geospatial (GIS) domain to develop 43 pollution scenarios contributing to the awareness and environmental consciousness of trainees and pupils (Fig. 1a,b and Supplementary Table S1). The locations and types of pollution scenarios were hypothetical, but real oceanographic and meteorologic data spanning from 2018 to 2019 were used in their computation.

Bathymetric data from EMODNET^15^ and Digital Elevation Models (DEM) digitised from topographic maps published by the Hellenic Army Geographical Service (H.A.G.S.) were used to: (a) improve our understanding of how seafloor morphology affects the dispersion of marine pollution (oil spills and floating debris), as bathymetric features are able to alter ocean circulation^16–18^, and (b) highlight the contribution of the onshore topography in a selected estuary subject to environmental pressures (climate change, agriculture, urban activity, tourism). The methodology followed to process the bathymetric data took into account the slopes and aspects, as well as the derivatives of bathymetric/elevation data to compute the lineaments present on Earth’s surface, either offshore or onshore^19^. A large part of these lineaments is due to the geodynamic activity that severely contributes to the past and present shape of Earth’s surface. High-resolution topographic data at 1 m contour intervals from the Geropotamos River, Messara Basin (Central Crete, Greece), were also used to show the contribution of morphometric analyses (slope map, aspect map, curvature of the slope map, flow accumulation map) to the development of estuary morphological models—these are crucial to address coastal protection issues (Supplementary Figure S1). The processing of the elevation data was computed using ArcGIS^20^.

In a second phase, the software packages GNOME^21^ and ADIOS^22^ were used to replicate oil spills, chemical releases and floating objects under particular meteorologic and oceanographic conditions. Pollutant dispersion was modeled using GNOME (General NOAA Operational Modeling Environment), a software tool used to predict the possible route, or trajectory, that a pollutant might follow in or on a body of water (see Supplementary Movie S1). NOAA's (National Oceanic and Atmospheric Administration) stand-alone oil weathering model, ADIOS^21^, was also used to understand the fate of oil when combined with high resolution data for wind, wave, sea surface temperature and 3D sea current conditions provided by the Oceanography Centre of the University of Cyprus^23^. Other physical parameters were obtained from GOODS (GNOME Online Oceanographic Data Server), an online tool that helps users access base maps and publicly available ocean currents and winds from various models and data sources^24^. In the scenarios that took into account oil or fuel spills from ships, fate models were computed to estimate the percentage volumes of oil evaporated, dispersed, and at the surface of the sea, after a certain number of days. Distinct volumes and densities of oil were considered in the models, from heavy bunker fuel to light oil with lower density. Floating objects such as plastic were modeled in a similar way, with an example of such modeling shown in Supplementary Figures S2 and S3.

In order to illustrate how the magnitude of flooding events may change in the future, we used climate impact indicators generated by the Service for Water Indicators in Climate Change Adaptation (SWICCA) project, a proof-of-concept developed for the Sectorial Information Service on Water Management for the Copernicus Climate Change Service (C3S). The indicators are based on hydrological simulations using the pan-European hydrological model E-HYPE^25^, with input variables from four (4) RCM simulations (Supplementary Table S2), corrected for biases and spatially interpolated as part of the EU FP7 project IMPACT2C. Several different indicators are available on gridded and catchment-scale resolutions. Because we are interested in high rainfall events leading to significant river discharge, we chose to use the indicator ‘flood recurrence’, representing the daily flow (m^3^/s) that corresponds to flooding events with 2, 5, 10, 50 and 100 years return periods, as well as a metric representing the average seasonal ‘river flow’ (m^3^/s). To maximise the climate change signal, indices representing the high emission pathway RCP8.5 for a far-future time horizon (2071–2100 relative to 1971–2000) were selected in this work. SWICCA indicators were retrieved for 10 of the 43 pollution scenarios, selecting for each location three catchments adjacent to the incident site (Fig. 1b). Except for sites ‘Cornwall’ and ‘Central Aegean’, the three selected catchments are located near the accident scenarios shown in Fig. 1b.

### Pedagogical approach

The promotion of marine and ocean conservation was considered, for the purposes of the Sea4All project and this work, a matter of changing human behaviour that can only be achieved through fostering a different frame of mind, a new ethos, and a new culture. Sea4All considered these latter aspects to be only achievable if built from a very early age, and emphasised multiple times through someone’s life. The key aim of the project was therefore to provide the teaching community with a complete educational kit on marine pollution, allowing teachers and pupils to investigate marine pollution issues in school, and outdoors, in interactive, creative, and experimental ways, assisted by innovative pedagogical Information and Communication Technology (ICT) tools.

To support the aims of the project, the Sea4All team developed a training curriculum aimed at teachers. The training curriculum was used to promote the professional development of teachers in the issues of marine protection and conservation, focusing on aspects as important as:

- Building a strong marine environmental awareness and consciousness in teachers and pupils;
- Enhancing the pedagogical competences and skills of teachers and educators in what the marine environment is concerned;
- Increasing pupils’ motivation and engagement in class, fostering their critical thinking;
- Adopting learning techniques that embrace the effective use of Information and Communication Technologies (ICT);
- Developing tools and e-learning materials focusing on key marine pollution issues;
- Building a creative and positive learning environment;
- Enhancing teachers’ knowledge on marine environmental issues through the implementation of Education for a Sustainable Development (ESD) competences.

An additional element of the project was to enhance the efforts for achieving Sustainable Development Goals (SDG) with emphasis on conservation of seas and sustainable use of marine resources. The training curricula was developed by the Unit of Education for Environment and Sustainable Development of the Cyprus Pedagogical Institute, in collaboration with the remaining Sea4All project partners.

## Results

### Analysis of pollution sources

The impacts of marine pollution have been extensively covered in the *mass media* for the past few years and are mostly felt around countries close to busy shipping lanes, or in heavily populated coastal areas. Floating objects and all types of chemical pollutants can impact marine ecosystems and water quality to affect coastal populations both economically and socially. In our analysis, we classified main types of marine pollution as follows:

#### Flotsam, jetsam and litter

**Figure 2:**
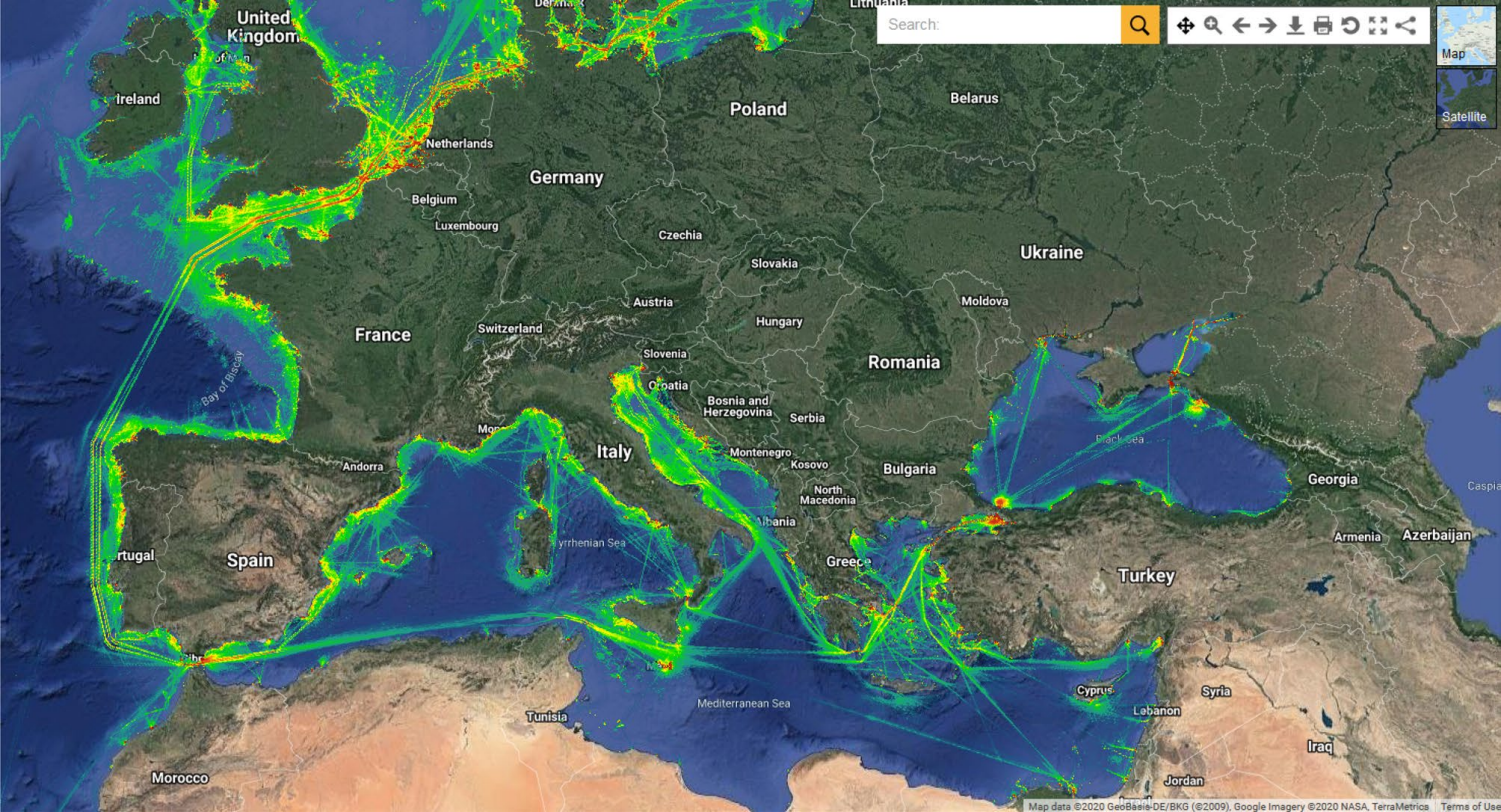
Ship density map for the seas surrounding Europe for 2018. The map illustrates the high density of vessels in confined maritime areas. The brighter colours indicate the presence of shipping corridors where ship density is the greatest. Map was taken from EMODnet data visualisation portal at https://www.emodnet-humanactivities.eu/view-data.php.

A first example of a major source of floating debris (and oil) comprises commercial shipping. Figure 2 depicts a heat map of ships crossing the European seas every year^26^—warm yellow/green and red colours represent the busiest shipping corridors around Europe. Ships sailing through international waters usually comprise large cargo ships and oil tankers interspersed with smaller fishing boats, recreational vessels, cruise ships, military ships, tug boats and scientific vessels. Figure 2 reveals the busiest shipping routes to be offshore Sicily (Southern Italy), West Iberia (Portugal, Spain) and in the English Channel. They highlight natural bottlenecks for marine traffic heading to, or leaving, ports in Europe.

In the broader Eastern Mediterranean and Black Sea regions, the Aegean Sea and the area around the Suez Canal are very busy shipping routes (Fig. 2). The sea around the Suez Canal comprises the second busiest route in the world, second only to the Malacca Straits offshore Malaysia^27^. Important shipping volumes also cross the Bosporus Strait into the Black Sea, via the Aegean Sea (Fig. 2).

Large ships are important sources of flotsam and jetsam. They are also important sources of illegal oil spills released by tankers sailing in international waters^28^. Moreover, the impact of maritime accidents increases significantly when involving large vessels, particularly when larger volumes of bunker fuel, chemical or oil loads are spilled.

#### Plastic, litter and chemical spills disposed of in rivers and streams

**Figure 3:**
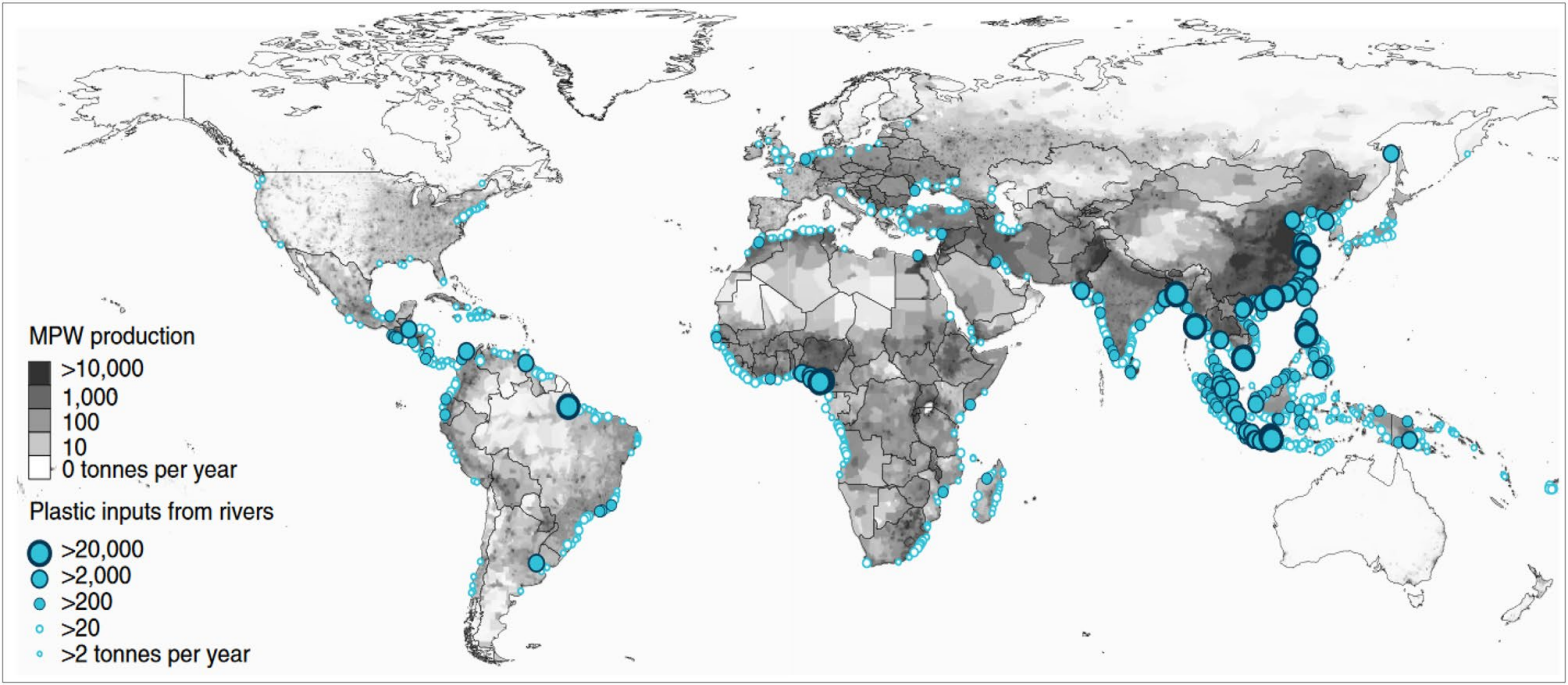
Map illustrating the mass of river plastic flowing into oceans in tonnes per year^29^ in different parts of the world. River contributions were estimated from individual watershed characteristics such as population density (in inhabitants per km^−2^), mismanaged plastic waste (MPW) production per country (in kg inhab^−1^ day^−1^) and monthly averaged run off (in mm day^−1^). The model^29^ is calibrated against river plastic concentration measurements from Europe, Asia, North and South America.

Rivers are an important source of pollution. It is known that 95% of the plastic in the sea comes from 10 rivers located in industrialised and heavy populated regions^29^ (Fig. 3). The most polluting rivers are located in Southeast Asia, close to the South China Sea, and in India, Bangladesh and Indonesia, all of which flow through large metropolises^29^. Other rivers releasing important volumes of litter at present are located in Africa; the Nile River that crosses Cairo, and the Niger River crossing Lagos and large cities in southern Nigeria, and in South America (Amazon River, Fig. 3).

Apart from the Nile River flowing from North Africa and ending in the Eastern Mediterranean Sea, there are other significant sources of pollution in Europe. The Danube River is historically a source of pollution, reaching Romania after crossing ten (10) countries on its way to the Black Sea^30^. The Dnieper and Don Rivers crossing the Ukraine and Russia are important waterways for shipping traffic and industry, flowing into the Black Sea. Fertilizer use and its illegal disposal near major rivers is an issue faced by agricultural areas of Eastern Europe, contributing to the eutrophication of rivers and lakes^31^. Further west, the Po (Italy), Rhone (France) and Ebro (Spain) are important rivers with significant sources of pollution^31,32^. Rivers such as the Tagus (Portugal, Spain), Thames (UK) and Seine (France) recorded important industrial pollution in the past century^33^.

#### Cultural events

**Figure 4:**
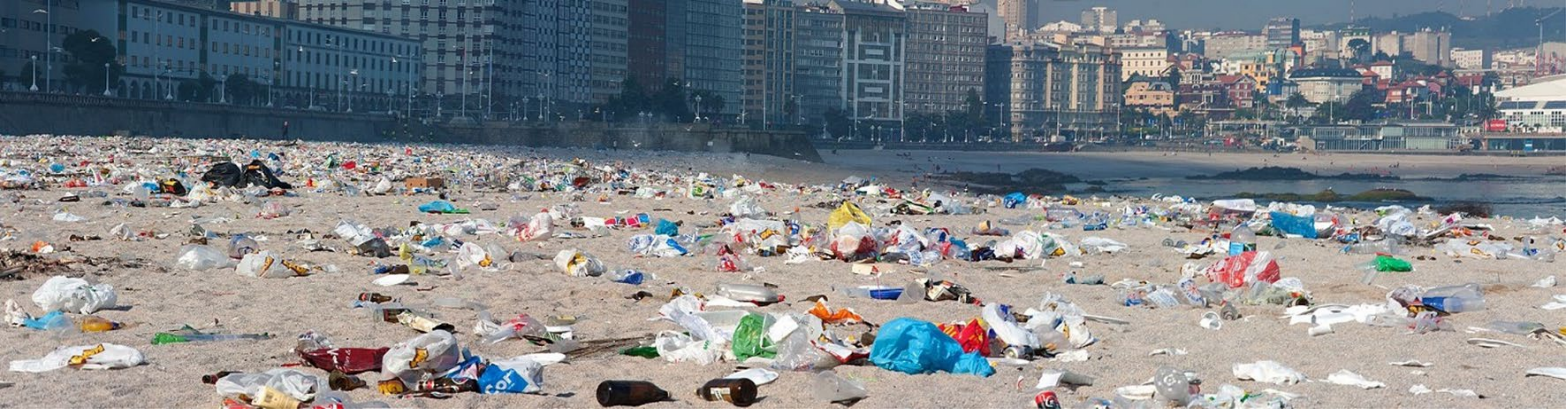
Panoramic photograph of the beach front at La Coruña, Spain, showing the impact cultural events may have on the coast. The image, highlighting the large amount of litter gathered on the Orzán Beach after the city’s Patron Saint celebrations (https://commons.wikimedia.org/wiki/File:Estado_de_la_playa_del_Orz%C3%A1n_despu%C3%A9s_de_la_noche_de_San_Juan_-_A_Coru%C3%B1a,_Galicia,_Spain_-_24_June_2010.jpg) was modified from an original photo taken by Carlos de Paz, an inhabitant of the city of La Coruña, on 24 June 2010. The image is licensed under the Creative Commons Attribution-Share Alike 2.0 Generic license, https://creativecommons.org/licenses/by-sa/2.0/.

Cultural events organised near the shore can potentially pollute vast areas, particularly in places where crowds are uncontained and litter cannot be quickly (and systematically) collected. Known examples of cultural events with remarkable numbers of attendants include the Rolling Stones’ music concert held in Copacabana (Rio de Janeiro) in February 2006, in which 1.5 Million spectators were present, and the 2013 mass delivered by Pope Francis on this same beach, attended by over three Million people^34^. Both these events benefited from litter-collection operations after they were held. Another remarkable example shown in Fig. 4 is from La Coruña, in Northern Spain, and shows the main beach of the city after the 2010 celebration of its Patron Saint, John. The presence of several thousands of people on the beach led to the accumulation of litter on the beach front, some of which was certainly washed out to sea at high tide.

#### Fishing gear and ghost nets

**Figure 5:**
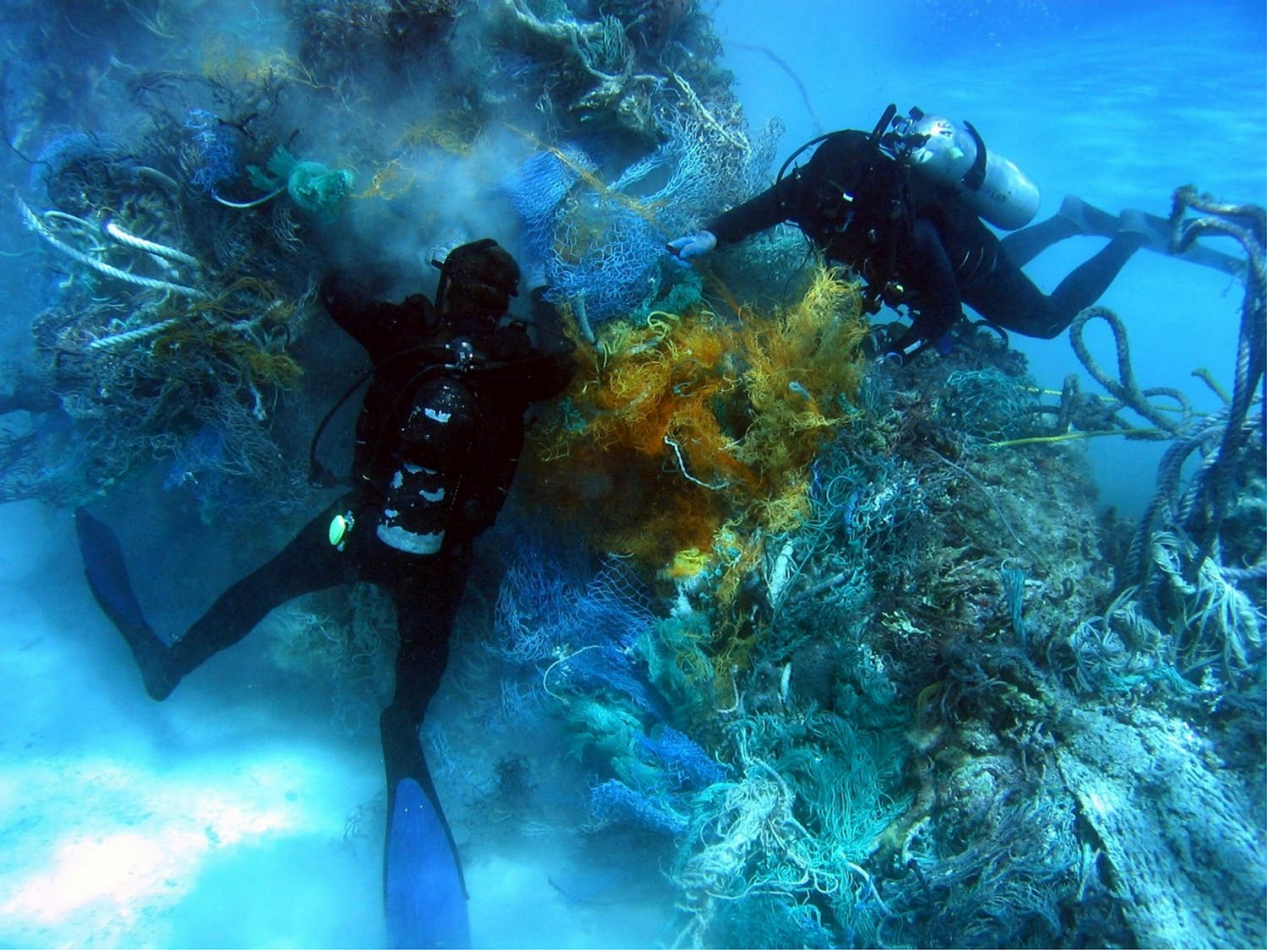
Underwater photograph showing Scuba divers removing derelict nets from a seafloor reef off NW Hawaii, United States of America. Water depth reaches 30 feet (~ 9 m) around this reef. After being swept by the North Pacific Gyre, a number of nets were bundled together to form a debris pile at this location. The photograph was taken by Dr. Dwayne Meadows, on 21 October 2010, is part of NOAA’s Photo Library (https://commons.wikimedia.org/wiki/File:Reef3038_-_Flickr_-_NOAA_Photo_Library.jpg). It licensed under the Creative Commons Attribution-Generic 2.0 license at Wikipedia Commons https://creativecommons.org/licenses/by/2.0/.

A final example of an important source of plastic pollution are ghost nets, i.e. fishing nets trapped on seafloor rocks and outcrops that are capable of capturing sea life for a large period of time (Fig. 5). In some instances, they continue to fish for months after they are trapped on the sea bottom. These nets systematically release fragments of plastic when breaking down naturally. The photo in Fig. 5 is from Hawaii, in which divers are contracted to clean some of these bundles, floating and towing some of the nets towards the surface.

### Bathymetric and elevation analyses

**Figure 6:**
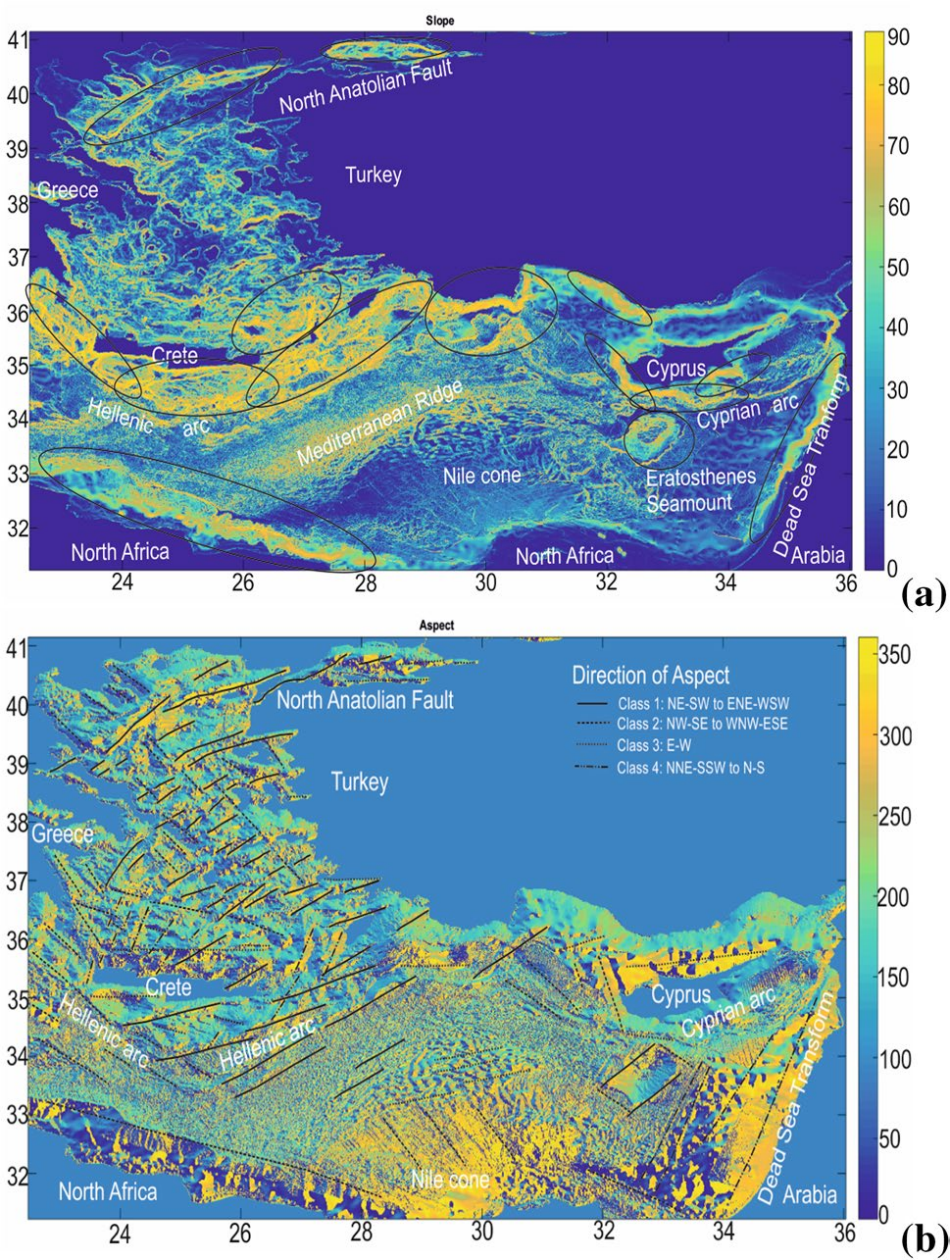
Example of bathymetric analyses for the Eastern Mediterranean Sea showing: (**a**) slope gradients and (**b**) aspects. Areas presenting slopes higher than 60° are indicated by the ellipses in (**a**), while aspects have been categorised according to the direction of aspect in (**b**), aiming at highlighting the role of the aspect direction in shaping the structure of the modern sea floor^35^. Seafloor topography severely alters the ocean circulation, especially in areas with rugged sea floor, further affecting pollution dispersion^36–38^. The Figures were compiled from Matlab-r2010a (http://matlab-r2010a.software.informer.com/). Bathymetric data are from the open database EMODnet (http://www.emodnet-hydrography.eu/).

The majority of the lineaments on Earth’s surface are related with geological faulting that is one of the most important factors responsible for the present shape of Earth’s terrain. Seafloor morphology strongly contributes to the expansion of marine pollution because it is able to alter the movement of sea currents^16–18^. In Fig. 6 we present an example of bathymetric analyses from the area of Eastern Mediterranean, aiming to highlight the role of slopes (already known) and aspects in seafloor morphology.

Morphometric analyses comprise an important tool to describe the physical characteristics of a drainage basin as a function of soil erosion patterns, runoff processes and water/pollution distribution. An example of morphometric analyses (elevation map, slope map, aspect map, curvature of the slope map and flow accumulation map) from the estuary and part of the catchment of Geropotamos River in Messara Basin (Central Crete, Greece) is shown in Supplementary Figure S1. Flow accumulation is strongly related to elevation, slope, aspect and curvature of the slope in the present case. The flow accumulation map corresponds to the accumulated flow and it comprises a useful tool to predict the locations of floating debris and chemical pollutants in case river flows increase significantly.

### Climate change scenarios

In order to understand the impact of climate on marine pollution sources, we used the SWICCA database of hydroclimate indices to analyse the impact of predicted climate change on monthly mean flow and flood recurrence magnitudes of a selection of catchments. These catchments were geographically distributed and climate change predictions were made for 2080 against a reference time period between 1971 and 2000 (Fig. 1b and Supplementary Table S2). Ten of the 43 incident sites were used to illustrate how climate change can influence patterns of discharge in European rivers in decades to come (Fig. 1b).

**Figure 7:**
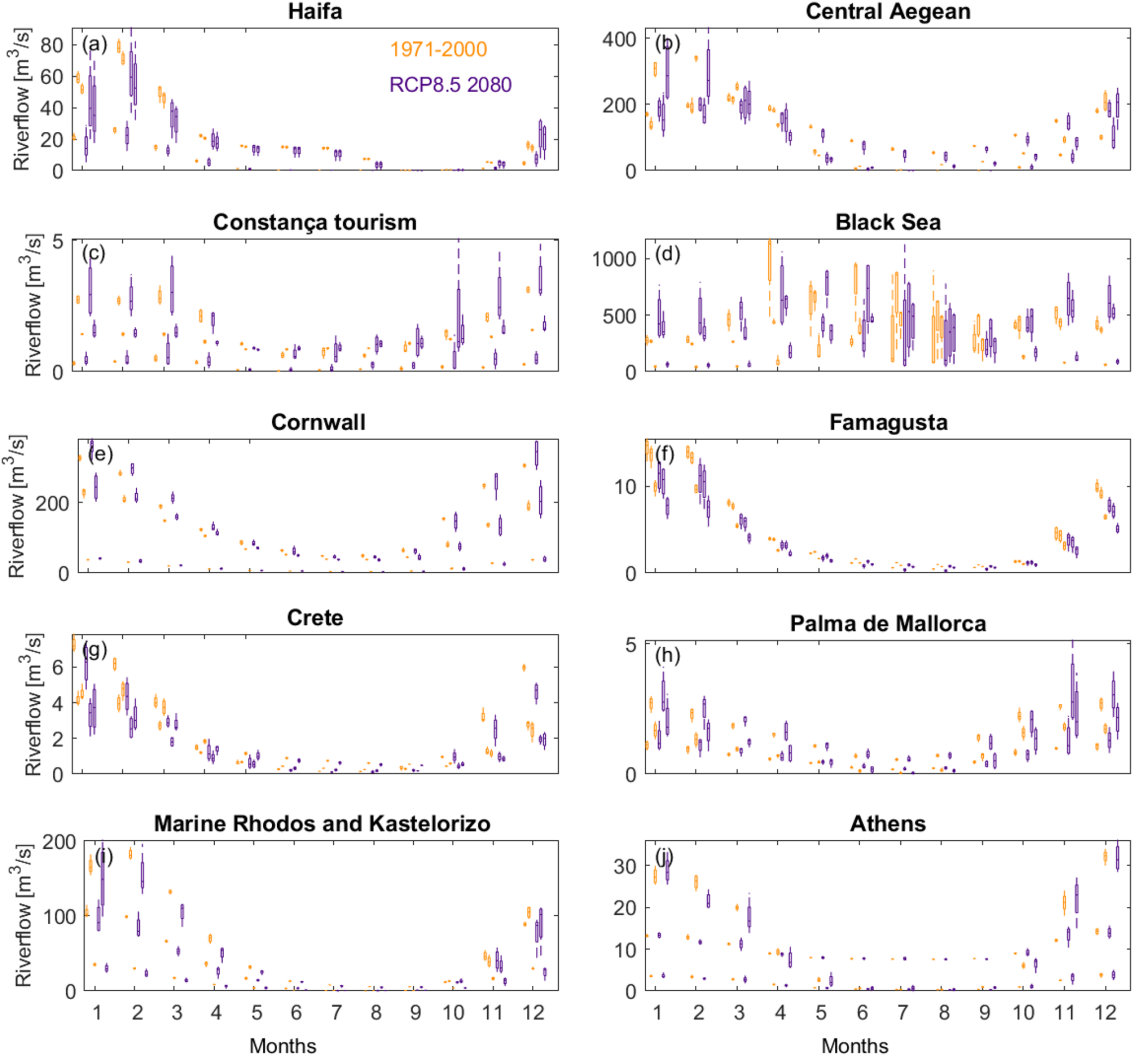
Bar graphs of simulated mean monthly flow (m^3^/s) for three catchments at each selected incident sites (see Fig. 1b for location). Orange colours represent discharge for a current time horizon (1971–2000). The indigo coloured bars represent discharge for a 30-year time horizon in the future, centered on 2080 following the RCP8.5 emission scenario. Most locations represented in this figure record a drier summer half-year compared to the winter half-year (November to March). Estimates of future change indicate much uncertainty, sometimes indicating that both decreases and increases are plausible futures. Graphs suggest a drying signal in the east of Mediterranean, particularly clear for Haifa and Famagusta. Further to the west and north, the change-pattern in catchment river flow is largely reversed, with increases more likely in the wetter winter months.

Most catchments have a drier summer half-year (April to October) compared to the winter half-year (Fig. 7). Exceptions to this rule are the Haifa catchment in the Eastern Mediterranean, which shows a relatively shorter wet period (January to March), and the Black Sea catchments with increases in flow in April due to snowmelt. Projected relative changes of mean monthly river flows (m^3^/s) and flood recurrence show that climate change can impact different aspects of flow, noting that the ensemble spread in many catchments indicates large uncertainty in projected change (Fig. 7). Nevertheless, the model spread indicates a future increase in flood magnitudes (more so when considering the rarer events) for most sites considered in this work, even if mean flow shows little change or, ultimately, decreases. The exception is again Haifa, Eastern Mediterranean, where extremes (as well as mean river flow) are projected to decline in most models. Few catchments show a clear change in climatic signal.

The Eastern Mediterranean sites of Crete and Famagusta, followed by other Eastern Mediterranean sites, show progressive drying conditions in the models, but with marked variability during the summer months and, in some cases, during the winter months (Haifa, Central Aegean, Athens, Marine Rhodes and Kastelorizo) (Fig. 7). Small changes to mean river flow are expected for the Cornwall catchments, with more models simulating drying rather than wetting conditions in summer and autumn; a similar pattern to that of Palma de Mallorca. In this latter city, the large percentage changes are due to the overall very small discharge values in the catchments chosen for this site (Fig. 7).

Altogether different patterns of change are shown for the two Black Sea sites: Constança and Black Sea. In the former, more models simulate an increase than a decrease in mean river flow for most months, noting that small absolute values in flow cause even small absolute changes to appear as large variations. To the East, model simulations for the Black Sea catchments generally show an increase in mean river flow in winter and a decrease in late summer and early autumn. Increases in flow are also shown for extreme flood events, though in relative terms less than those projected for the Constança tourism site.

**Figure 8:**
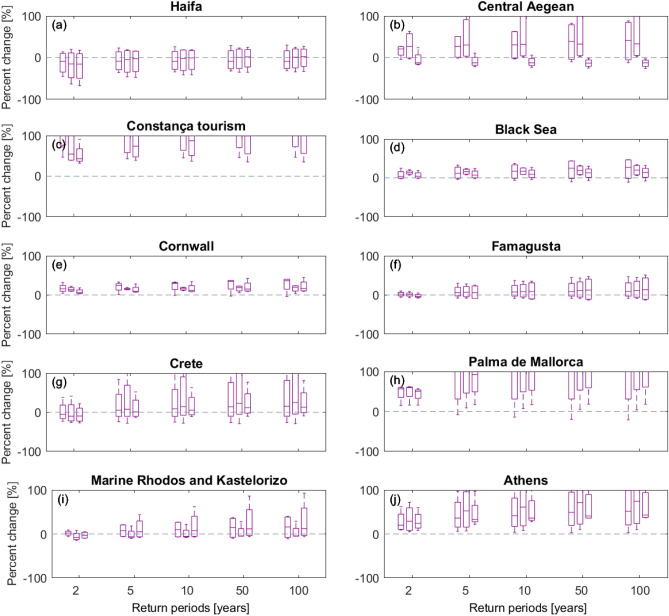
Bar graphs of relative change (%) in simulated annual flood frequency magnitudes for different return periods for three catchments for each selected incident sites. Relative change is estimated as the percent difference between indices estimated for a current climate period 1971–2000 and those for a 30-year period centered on 2080 following emission scenario RCP8.5. Axes are cut at 100% change to improve readability. Flood magnitude is predicted to increase in Athens, Palma de Maiorca and the Aegean Sea as a whole (including Crete) until 2018, and similarly so for Constança. Minor increases are estimated for Rhodos and Kastelorizo, together with Famagusta in Cyprus and Cornwall (UK). Estimates for the Black Sea differ from those for Constança, indicating only a slight increase in flood magnitudes for all return periods.

Based on the graphs in Figs. 7 and 8, catchments representing the Black Sea site are likely to experience increased mean flow, particularly in winter months; noting that there is much uncertainty depending on the selection of catchment and underpinning RCM. In contrast, Crete, Athens and the coasts bordering Greece and Cyprus are more likely to see a moderate reduction in river flow, though graphs indicate greater disagreement on the direction of change during summer months compared to winter months. Selected catchments representing Palma de Mallorca (Spain) and Cornwall have a similar pattern of change, with increases appearing more likely in winter months whilst most ensemble members simulate decreases in summer months (Fig. 8).

**Figure 9:**
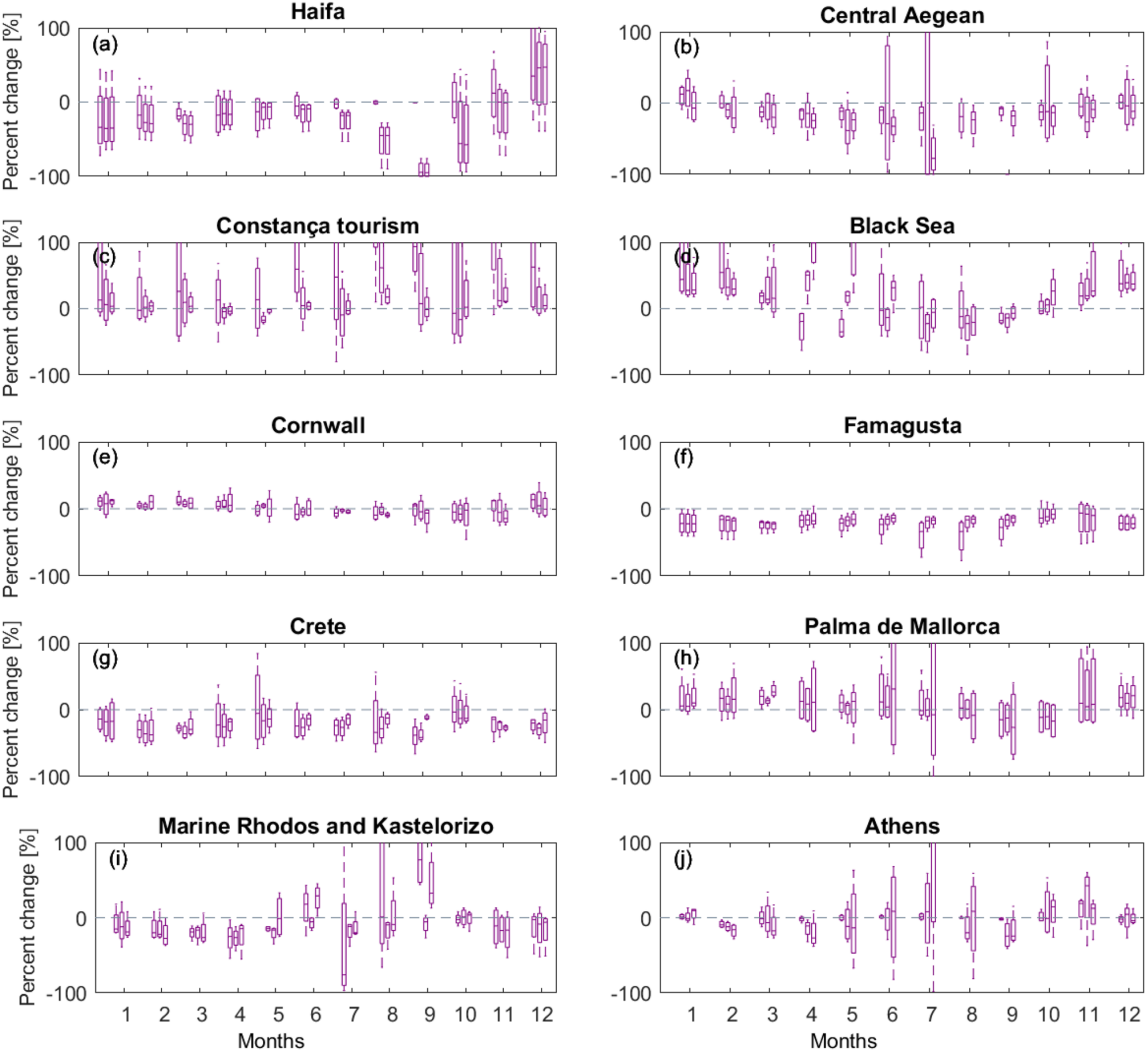
Bar graphs of relative change (%) in simulated mean monthly flow for different return periods for three catchments for each selected incident sites. Relative change is estimated as the percent difference between indices estimated for a current climate period 1971–2000 and those for a 30-year period centered on 2080 following emission scenario RCP8.5. Axes are cut at 100% change to improve readability. Monthly catchment flows are expected to decrease in Famagusta, Crete, Rhodos and Kastelorizo, with variations recorded amongst this locations. The Central Aegean is predicted to record drier summers but slightly wetter winters. The Black Sea and Constança will record increases in monthly catchment flow, likely in association with the capture of thawing water and river flow from surrounding mountains.

Whilst the patterns of change are somewhat different across the selected sites, most sites (with some exceptions, most notably all catchments around Haifa) suggest that the magnitude associated with flooding events is more likely to increase than decrease; more so for the more rare events (Fig. 9). These results are in agreement with previously published results on geographical patterns of hydroclimate variables in Europe^9^ and the expectation that rainfall intensities will amplify with atmospheric warming^34^.

## Discussion

### Impact of climate change on pollution sources

A key aspect of our analysis was to foresee if the climatic changes predicted for the 21st Century will impact on the types and volumes of pollutants sourced by Europe as a continent, with emphasis on the confined maritime areas of NW Europe, Mediterranean and Black Seas. Will changes in river flow and rainfall force one pollutant source over all the others, and will seasonality in weather replicate such changes?

Considering the variety of pollution sources identified in this work, the primary impact of climate change will be in the suppression (or imposition) of one pollution source in relation to the others. This means that, in case river flow significantly increases with time, rivers such as the Danube, Dnieper and Don in the Black Sea will become priority point-sources for floating debris and chemical pollutants, particularly after the dry summer season. In general terms, rivers are able to increase their volumes in a significant way after the summer season, remobilizing litter and chemical pollutants accumulated earlier in the year. We therefore predict an increase in the importance of rivers as a source of pollution in the areas where river flow (and rainfall) will increase significantly, such as in the northern regions of Europe. Conversely, the relatively drier regions around Greece and Cyprus will record an increase in flotsam and jetsam as sources of pollution, with rivers and streams contributing relatively less as pollution sources when compared to the present day (Fig. 9). A limitation to this postulate relates to the type and duration of river discharges—a relatively drier Eastern Mediterranean in 2080 may experience more frequent flash-flood events; i.e. large rain falls capable of transporting pollutants very quickly, and in very large volumes, within dense mixtures of sediment and water. In this latter case, if flash floods become more frequent, weather variability will closely influence marine pollution in Southern Europe.

A second major impact of climate change on marine pollution relates to its effects on weather seasonality^39^, i.e. how markedly different winter and summer weather conditions are expected to be in 2080, and beyond this date, considering the predicted increases in rainfall and river flow in the Black Sea, and the variable river flow predicted for Northwest Europe (Figs. 7, 8 and 9). The backdrop for these assumptions is that if winter and summer conditions become more marked, the tendency will be for rivers to contribute most effectively as point-sources of pollution during the first fall-winter rains, when flash floods are more intense and pollutants accumulated during the summer are easily remobilised. In contrast, the summer months will be dominated by flotsam and jetsam as main sources of pollution, together with the accidental release of litter and debris by tourists in popular beaches and cities. In fact, longer summer seasons will likely translate into a larger volume of tourists visiting Southern Europe, and larger amounts of recreational vessels in the Aegean and Mediterranean Seas*.*

A third expected impact of climate concerns farming as a main source of fertilizers, plastic debris and industrial litter to the European seas^29–32^. The Mediterranean has recorded, for the past two decades, one of the greatest increases in the use of greenhouses in farming, an aspect not limited to southern Europe as greenhouse farms are now also ubiquitous in SE Asia, India and the Middle East^40^. In regions such as Crete, SE Spain and South Italy, greenhouse use has contributed to important pollution in otherwise intermittent streams, in which litter is dumped in the summer and then washed downstream during flash-flood events. Fertilizers are also an important source of pollution here, as these same flash floods are capable of remobilizing large volumes of soil contaminated with fertilizers used in intensive farming practices^32,40^.

### A pedagogical method to teach marine pollution

Knowing in advance that marine and coastal environments are valuable parts of our planet, the Sea4All project aimed to build a strong environmental consciousness in school pupils primarily aged 10–14. We considered, as a key aspect of Sea4All, that changes in the pupils’ education should be nurtured from a small age, to be reinforced in a later stage during high school. We therefore created online education material, games and tools to promote the environmental awareness and consciousness in school pupils, and within the educational community (i.e., teachers and educators). More than 10 schools were involved and contributed to the project development in four (4) participating countries, United Kingdom, Greece, Cyprus and Romania. In addition, more than 400 users participated in the e-game and provided feedback.

In our pedagogical approach, the volume of marine traffic, as well as the most common types of vessels travelling through specific shipping lanes is considered first—it was systematically mapped in the Mediterranean Sea and around Europe (see Fig. 2). Different pollution scenarios were subsequently developed taking into account the more environmental susceptible areas of Europe, and known bottlenecks in terms of marine traffic. These scientific deliverables are interconnected and were designed to prepare:

- A comprehensive training curriculum for educators and teachers;
- An e-learning platform for teachers interested in learning about (and ultimately delivering lessons on) marine pollution;
- A new ICT-based learning game on marine pollution;
- A pedagogical handbook for students;

The training curriculum for teachers and educators includes five (5) modules, as described below.

#### Module 1: teaching competences

Module 1 is used to clarify what teaching competences are, their importance and how they can be acquired by teachers and educators. The general concepts of “competence”, “competencies” and “skills” are defined and clarified. Module 1 also explains why teaching competences are important in Environmental Education/Education for a Sustainable Development (EE/ESD) and how they are developed. Teaching competences are discussed in light of the United Nations Decade for EE/ESD as an important tool towards sustainability. EE/ESD competences are structured within the four pillars of quality education: (a) learning to live together, (b) learning to know, (c) learning to do, and (d) learning to be. The EE/ESD programmes’ contribution to the development of Sustainable Development Goals (SDGs) are explained at the end of the module. EE/ESD aims to develop competences in learners contributing to the efforts in accomplishing the 17 SDGs. Finally, indicative examples of models are presented that aim to define the EE/ESD competences in a coherent and comprehensive manner.

#### Module 2: teaching methods

This module analyses teaching techniques that are commonly used in Environmental Education programmes. Such techniques are chiefly experimental, student-centered and promote an active participation of learners. They aim at promoting awareness on environmental issues, knowledge, attitudes and skills, and they encourage a collective effort and cooperation. Key teaching methods developed in this module include: (a) brainstorming, (b) concept maps, (c) debates, (d) role-playing games, (e) ethical dilemmas, f) field studies, ((g) modelling, (h) WebQuest activities, and (i) bibliographic research. In Module 2, every teaching method is described and followed by examples of marine pollution derived from the 43 pollution scenarios considered in this work. Tables, diagrams, pictures, worksheets and proposed sources of information are provided to teachers and educators, so that they easily understand key teaching techniques and use them in outdoor or indoor activities.

#### Module 3: scientific content knowledge

Module 3 summarises the physical and chemical processes that can lead to the drifting and natural breakdown of marine pollutants. Different ways of mitigating marine pollution are presented, together with information of the ways spilt oil and floating objects move within and on water, later affecting sea life. The term ‘bathymetry’ is explained and its contribution to enhance marine pollution issues is discussed. Bathymetry, mathematical models, oceanographic and meteorological data are used where necessary to predict the movement of oil spills and the floating objects. These mathematical models were used in the e-game, Pedagogical Handbook and the teachers’ Training Curriculum.

#### Module 4: marine pollution—theoretical framework

Module 4 emphasises the scientific, economic and social impacts of marine pollution (oil and floating debris) on coastal populations all over the world. The main sources for generating plastic pollution around the world are classified, as shown in the previous sections of this work, in: (a) flotsam, jetsam and litter disposed of by sea crews and passengers; (b) plastic litter and chemical spills (including oil) that is directly disposed of in rivers and streams, (c) cultural events; and (d) fishing gear (ghost nets).These sources are analysed in Module 4 and characteristic examples related to marine pollution from all over the world are presented.

#### Module 5: thematic examples

Five different scenarios are used as primary case-studies in Module 5. They include:

- Romania plastic pollution
- Shetland Islands Spill (UK)
- Oil spill pollution in Cape Greko (Cyprus)
- Large volumes of litter released on Samos due to incoming refugees (Greece)
- Ghost nets in the Thermaic Gulf (Greece)

Each scenario is described, based on scientific data (ecological information, bathymetry, oceanographic and meteorological information, etc.), and has very focused learning objectives and underpinning components. Example activities are provided using specific teaching methods, and each activity is connected with various EE/ESD teaching competences—as described in Module 1—using the Rounder Sense of Purpose (RSP) Competences (2018) model. The examples provide all the necessary material for the educator to help students learn about marine pollution in an interesting and intriguing manner.

### E-game as an innovative method to teach marine pollution

The Sea4All team chose to use innovative methods in Information and Communication Technology (ICT) to divulge the key scientific, ecological and pedagogical aspects of the project to schools and the general population. Pupils in schools around the world are an on-line crowd with variable degrees of access to the worldwide web through personal computers and mobile devices. Therefore, an innovative on-line game was developed, and aimed at advancing the skills of pupils, and motivate them (in a playful manner) to adopt responsible behaviors^41^.

**Figure 10:**
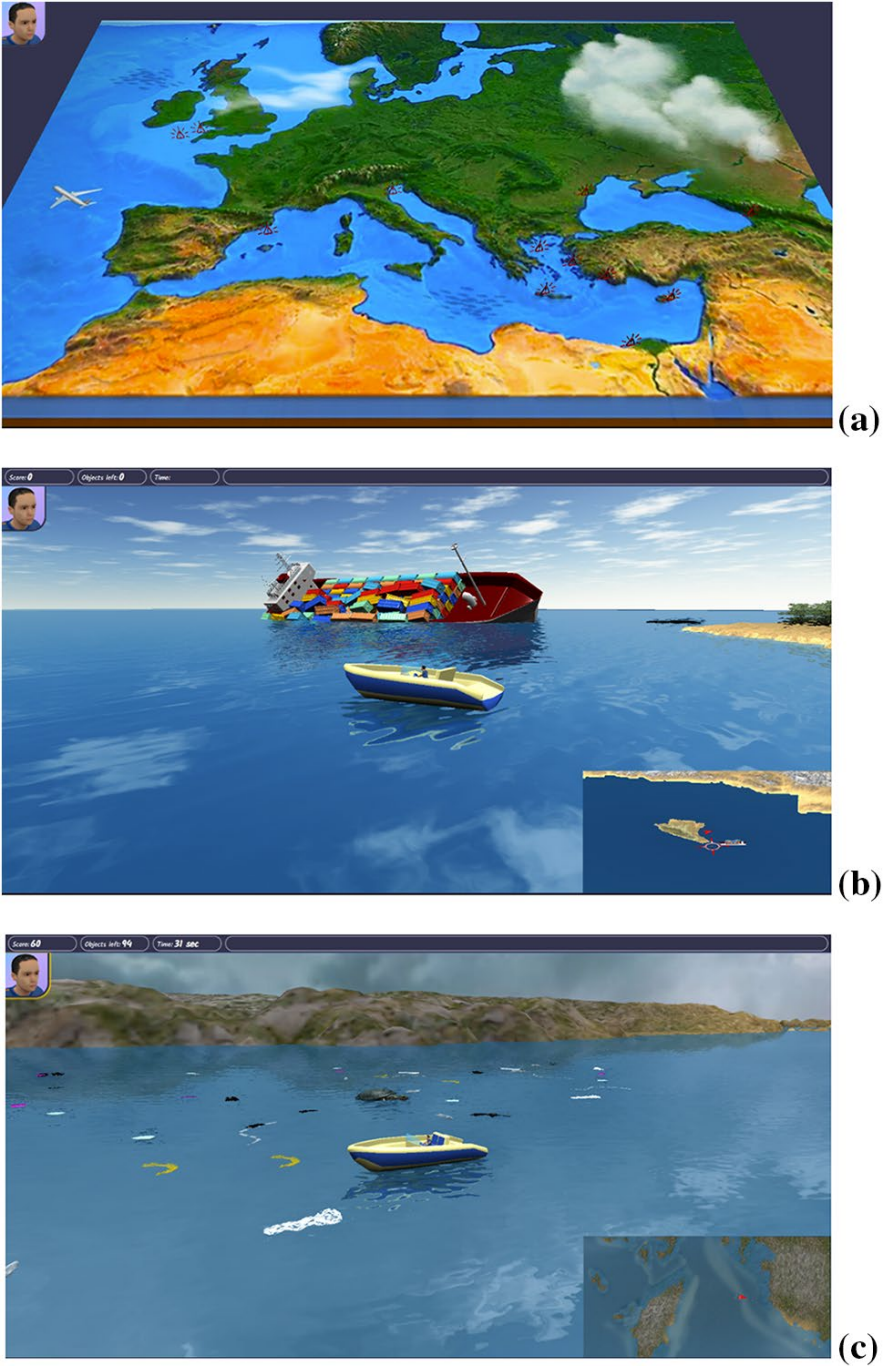
Screen images of the web-based game for PCs (using WebGL technology) compiled for the Sea4All project^41^. (**a**) General map showing the relative location of tasks to be completed by users, with rewards/bonus provided upon completion (e.g. inventory items, puzzles, new information about marine pollution, the possibility of unlocking new scenarios). (**b**) Example of a scenario in the game concerning the capsizing of a cargo ship and ensuing oil spill. The game users will have to confine the oil spill using equipment (booms) available on the imaged boat. (**c**) Example of a scenario that concerns the collection of marine litter close to an area with sea turtles. Note the presence of a moving sea turtle behind the main character’s boat, between this latter and the coast.

The game includes multiple scenarios, compiled for distinct meteorological and oceanographic conditions, simulating the movement of pollution in specific times of the year. In Fig. 10 are shown examples of the game when played, revealing tasks that pupils need to complete to end specific levels. The game includes a series of structured stories, or scenarios. The pupils will become familiar with a different way of learning, undertaking specific tasks in the game rather than listening or reading about marine pollution. Educational games such as this have the potential to be a significant tool for training and teaching as interaction with the learners is encouraged. For instance, the game benefited from the compilation of the novel oil-spill and floating-object pollution scenarios, and included 2D animations, the collection of maps and photos, videos and internet links (Fig. 10). Multilevel game-based learning, when combined with the teaching materials in the e-learning platform, provides the opportunity of transferring an integrated education in marine pollution issues, in a playful and reality-grounded way.

## Conclusions

A novel scientific, societal and education approach was attained by the Sea4All project, leading to a combined use of technical and scientific, technical and electronic information in the teaching of 10–14 year old pupils as priority age group. The main conclusions of this work are as follows:

1. Principal marine pollution sources in Europe, as well as in the worlds' seas, include: (1) rivers and streams near cities and industrialised areas, (2) coastal areas recording sharp demographic pressures during the tourism season, (3) offshore shipping lanes in which oil, flotsam and jetsam are released, and (4) areas of rough seafloor relief, where industrial fishing leads to the trapping of ghost nets.
2. Obtaining appropriate climate change information is critical as onshore pollution sources will likely vary in importance relative to climatic changes. We see different climate change signals in mean and extreme river flow, and marine pollution types will vary depending on seasonal and long-term climate change. At this point, we emphasise the role of morphometric analyses both onshore and offshore to assist in the prediction of locations where floating debris and chemical pollutants are accumulated.
3. In the Black Sea, larger river flows and less climatic seasonality are likely to result in more constant flows of marine pollution to the Black Sea. In contrast, a drier Eastern Mediterranean Sea are likely to result in discrete pollution episodes during seasonal flash-flood events, whilst drier summers will favor tourism, shipping (flotsam, jetsam) and fishing as major sources of pollution.
4. Our results have been used in the compilation of e-learning tools and e-games for teachers and pupils primarily aged 10–14 years, so that pupils, local populations and visitors to coastal areas are educated about their impact on the marine and coastal environments.

## Supplementary Information

Supplementary Information.

Supplementary Figures.

Supplementary Movie 1.

Supplementary Movie Legend.

Supplementary Table 1.

Supplementary Table 2.

## Data Availability

Relevant data and examples in this work are included as Supplementary Information and can also be requested using the contacts in the portal https://www.sea4all-project.eu/, where learning tools, curricula and scientific results are stored.
