# Supplementary material for: Scientific, societal and pedagogical approaches to tackle the impact of climate change on marine pollution
Source: Sci Rep. 2021 Feb 3;11:2927. doi: 10.1038/s41598-021-82421-y (PMC7858591; doi:10.1038/s41598-021-82421-y)

**Scientific, societal and pedagogical approaches to tackle the impact of climate change on marine pollution**

Tiago M. Alves^1,*^, Eleni Kokinou^2,3^, Marie Ekström^1^, Andreas Nikolaidis^4^, Georgios C. Georgiou^4^, Anastasia Miliou^5^

1) 3D Seismic Lab – School of Earth and Ocean Sciences, Cardiff University – Main Building, Park Place, Cardiff, CF10 3AT, United Kingdom ([alvest@cardiff.ac.uk](mailto:alvest@cardiff.ac.uk))

2) Laboratory of Applied Geology and Hydrogeology, Department of Agriculture, Hellenic Mediterranean University, P.O. Box 1939, 71004, Heraklion, Crete, Greece ([ekokinou@hmu.gr](mailto:ekokinou@hmu.gr))

3) Foundation for Research and Technology-Hellas, Institute of Computer Science, 70013 Heraklion, Crete, Greece

4) Oceanography Centre, University of Cyprus, P.O. Box 20537, 1678 Nicosia, Cyprus (and@ucy.ac.cy, georgios@ucy.ac.cy)

5) Archipelagos Institute of Marine Conservation, P.O. Box 42, Pythagorio 83 103, Samos, Greece

Supplementary Figure 1 – Example of morphometric analyses for the estuary of the Geropotamos River as well as part of its catchment in Messara Basin (Central Crete, Greece), using high-resolution elevation data of 1 m contour interval digitized by the topographic maps published by the Hellenic Army Geographical Service (H.A.G.S.). a) Elevation map in meters, b) slope map in degrees, c) aspect map in degrees, d) curvature of the slope map, e) flow accumulation map.

**
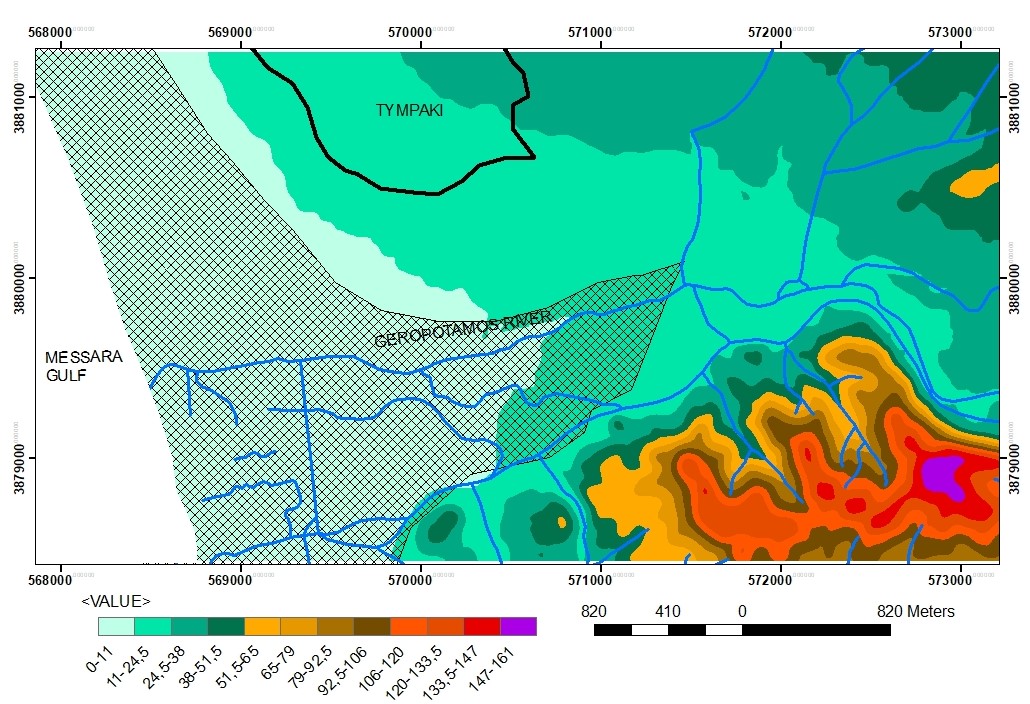
a)**

**
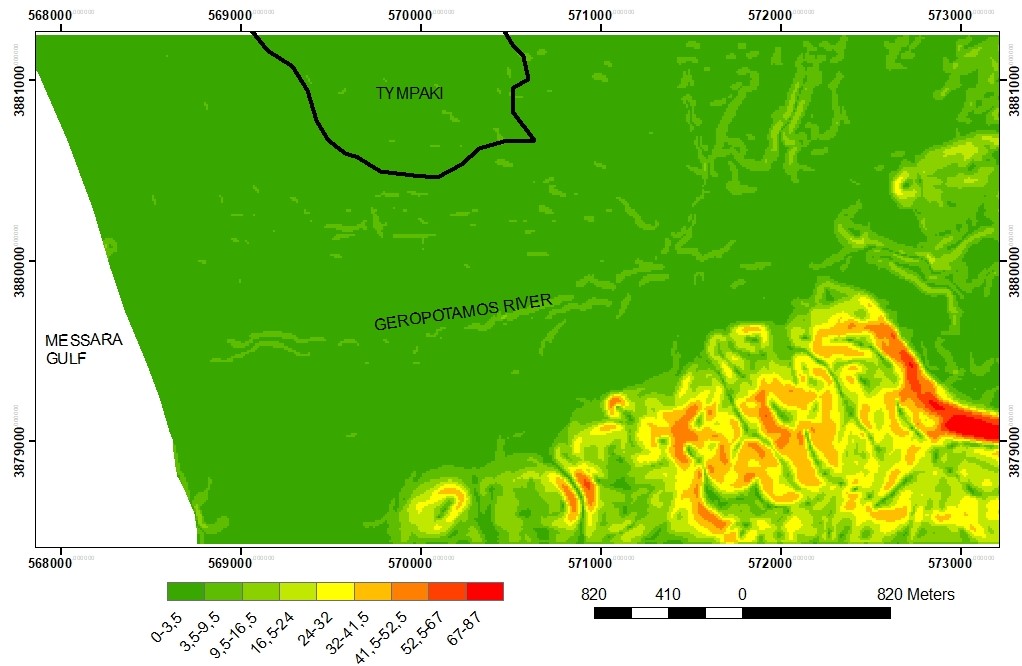
b)**

*
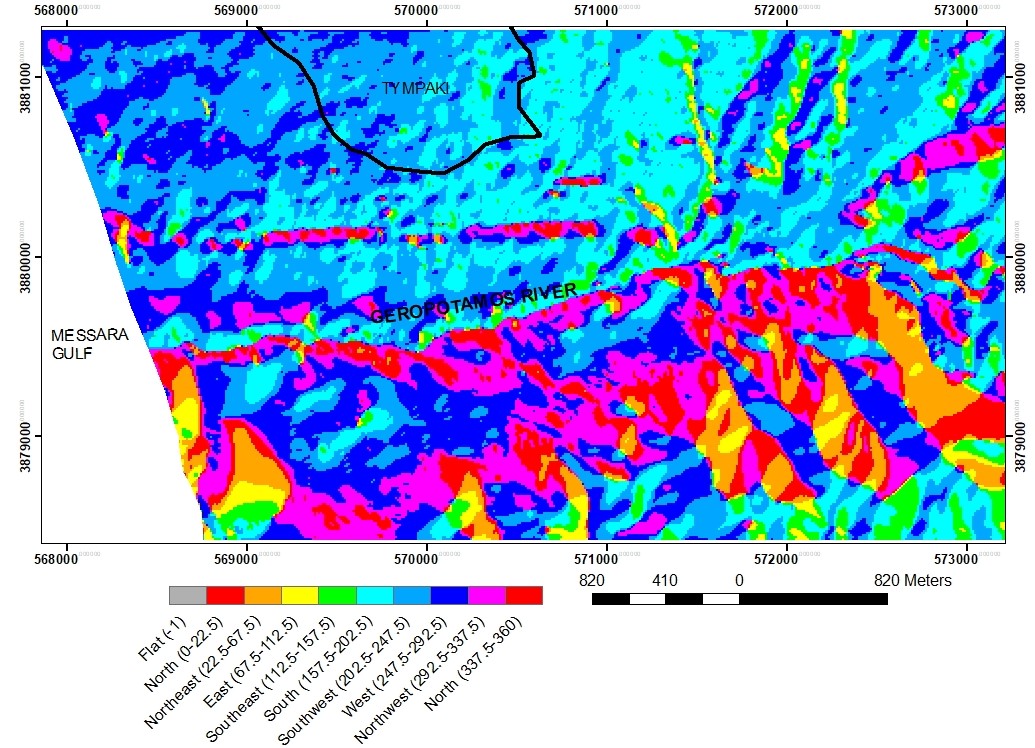
***c)**

*
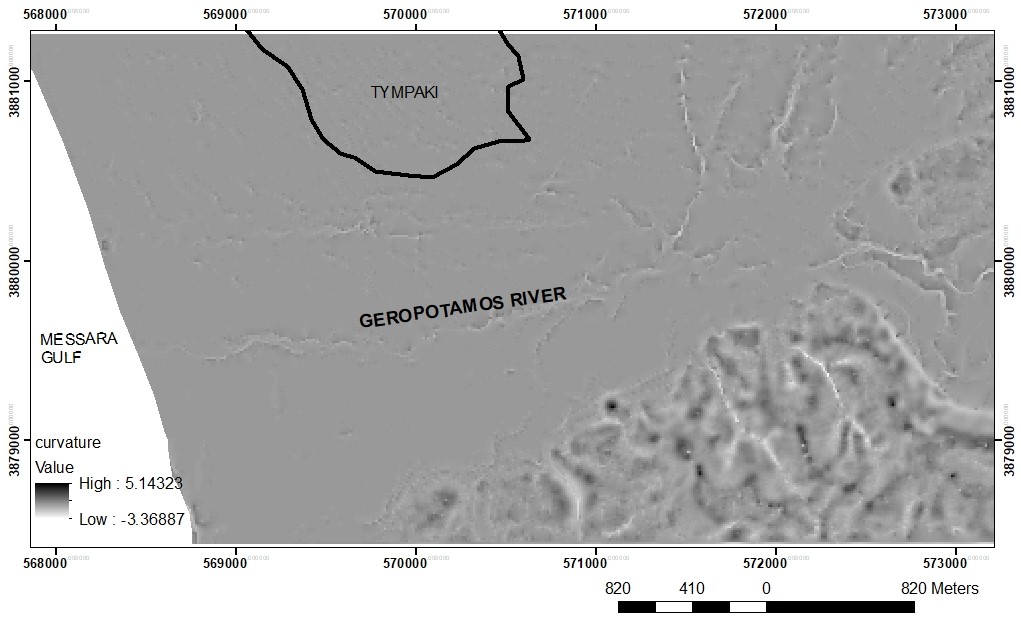
***d)**

*
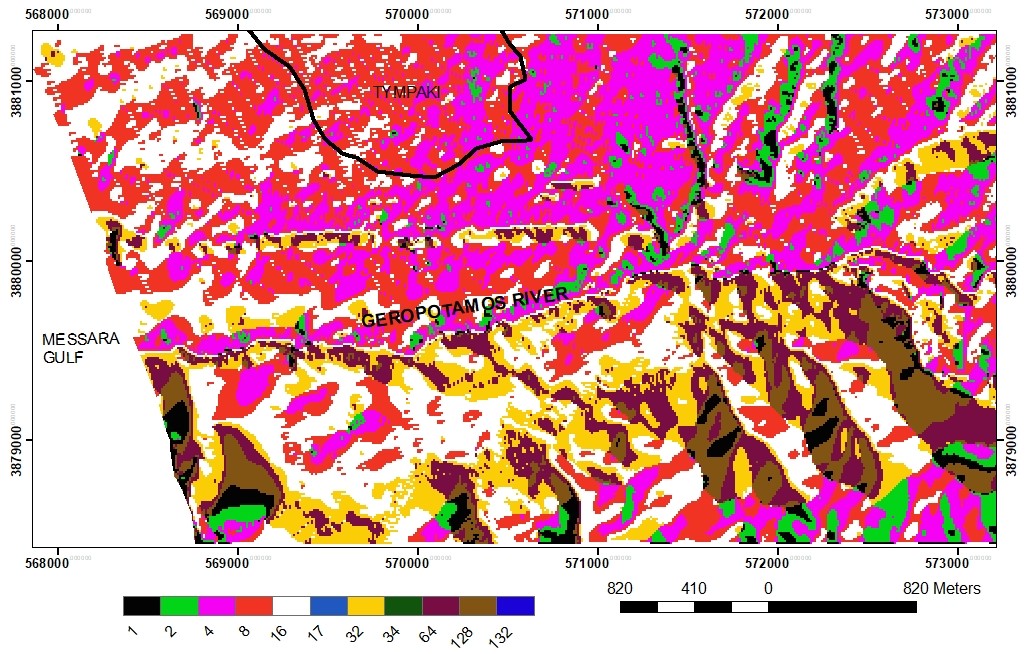
***e)**

Supplementary Figure 2 – Example of GNOME models for a location south of Gavdos Island, offshore Greece (Scenario 6 in Supplementary Table 1). a) Oil spill after 1 hour (23/01/2018 12:00 UTC). The wind is from the SW with an average intensity 20 m/s. Status: The remaining oil constitutes the 91% of the total released amount, while 7% was evaporated and 1% dispersed after 1 hour. b) Oil spill after 7 hours (23/01/2018 18:00 UTC). The wind is from the SW with an average intensity 20 m/s. Status: The remaining oil constitutes the 58% of the total released amount, while 40% was evaporated and 2% dispersed after 7 hours. c) Oil spill after 11 hours (23/01/2018 22:00 UTC). The wind is from the SW with an average intensity 20 m/s. Status: The remaining oil constitutes the 39% of the total released amount, while 58% was evaporated and 2% dispersed after 11 hours. d) Oil spill after 17 hours (24/01/2018 04:00 UTC). The wind is from the SW with an average intensity 20 m/s. Status: The remaining oil constitutes the 25% of the total released amount, while 73% was evaporated and 2% dispersed after 17 hours.


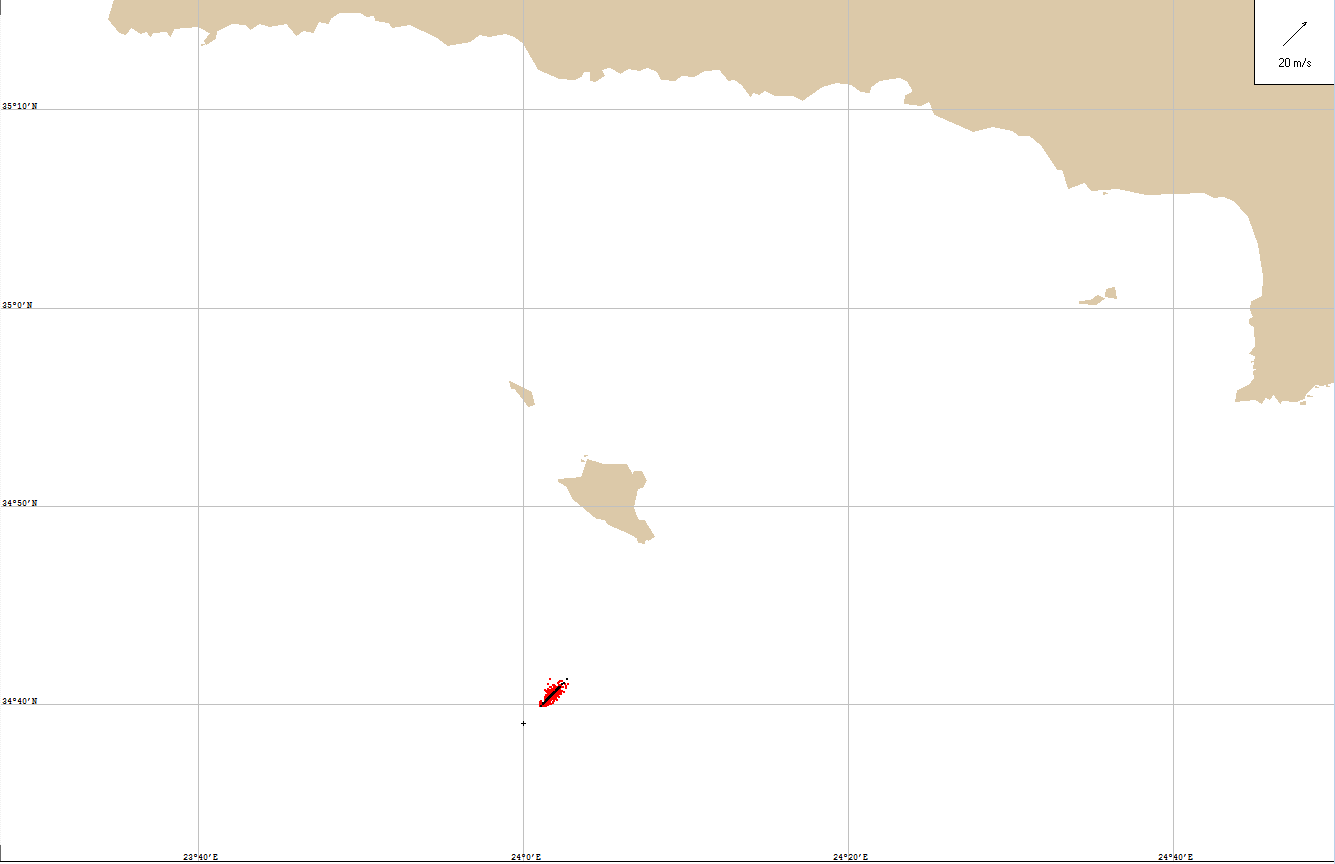
**a)**

**
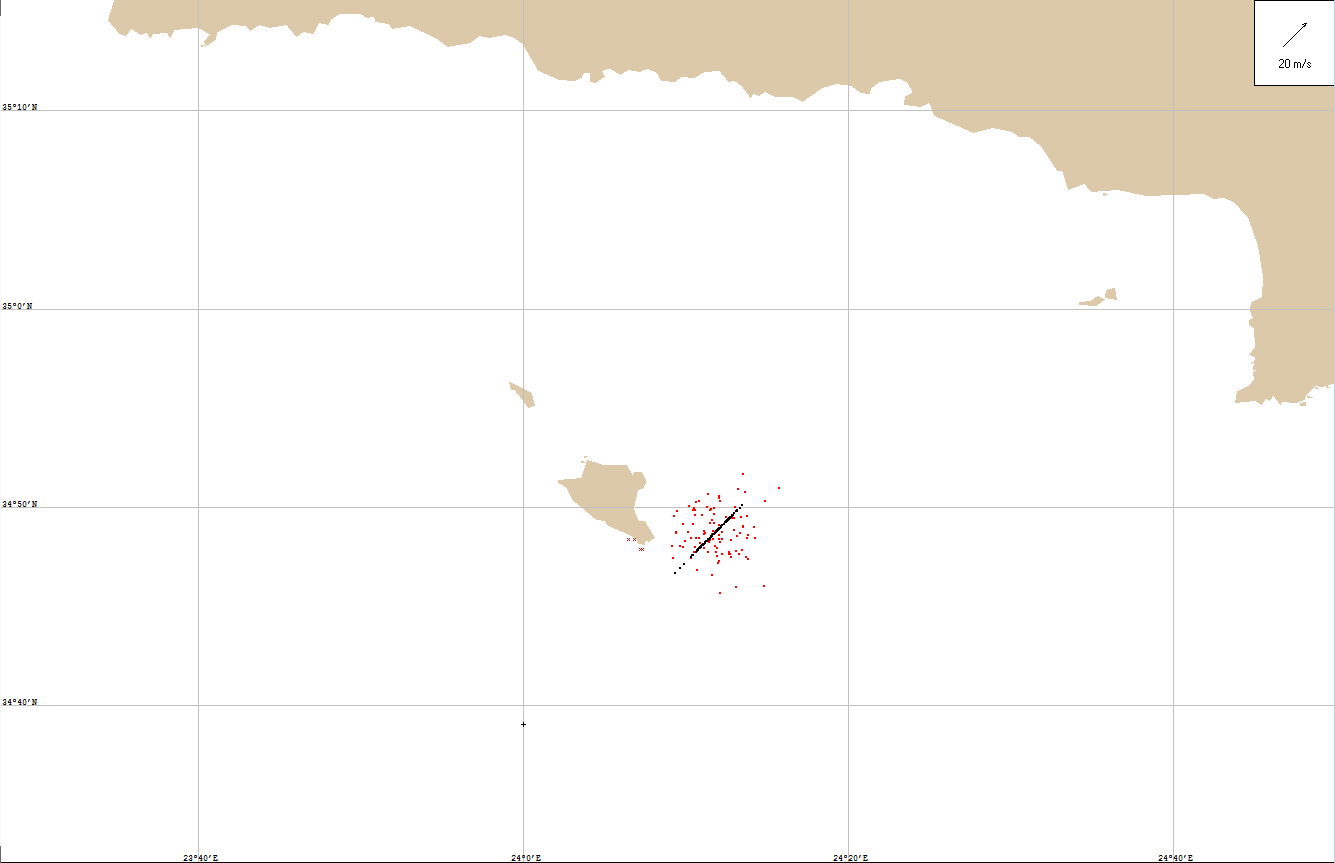
b)**


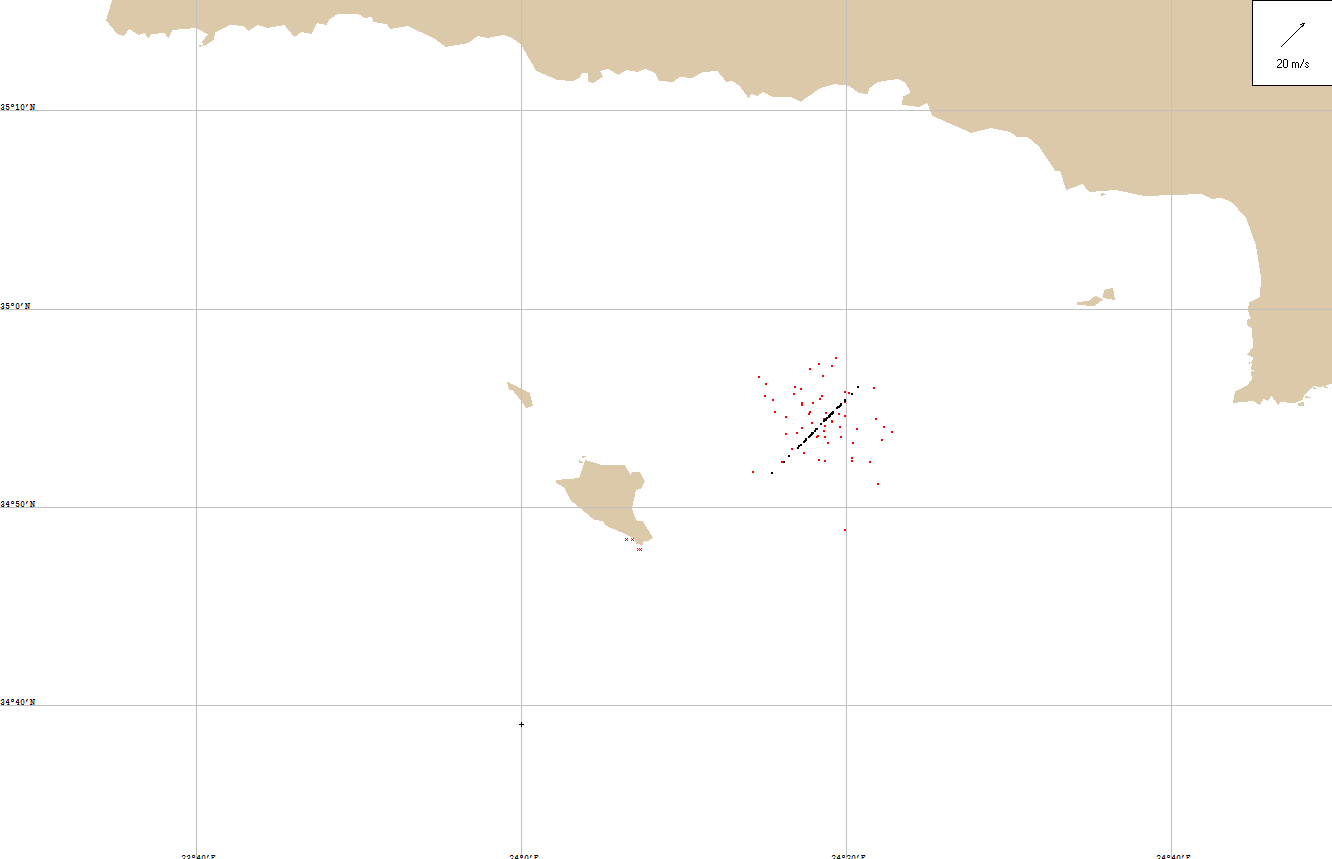
**c)**


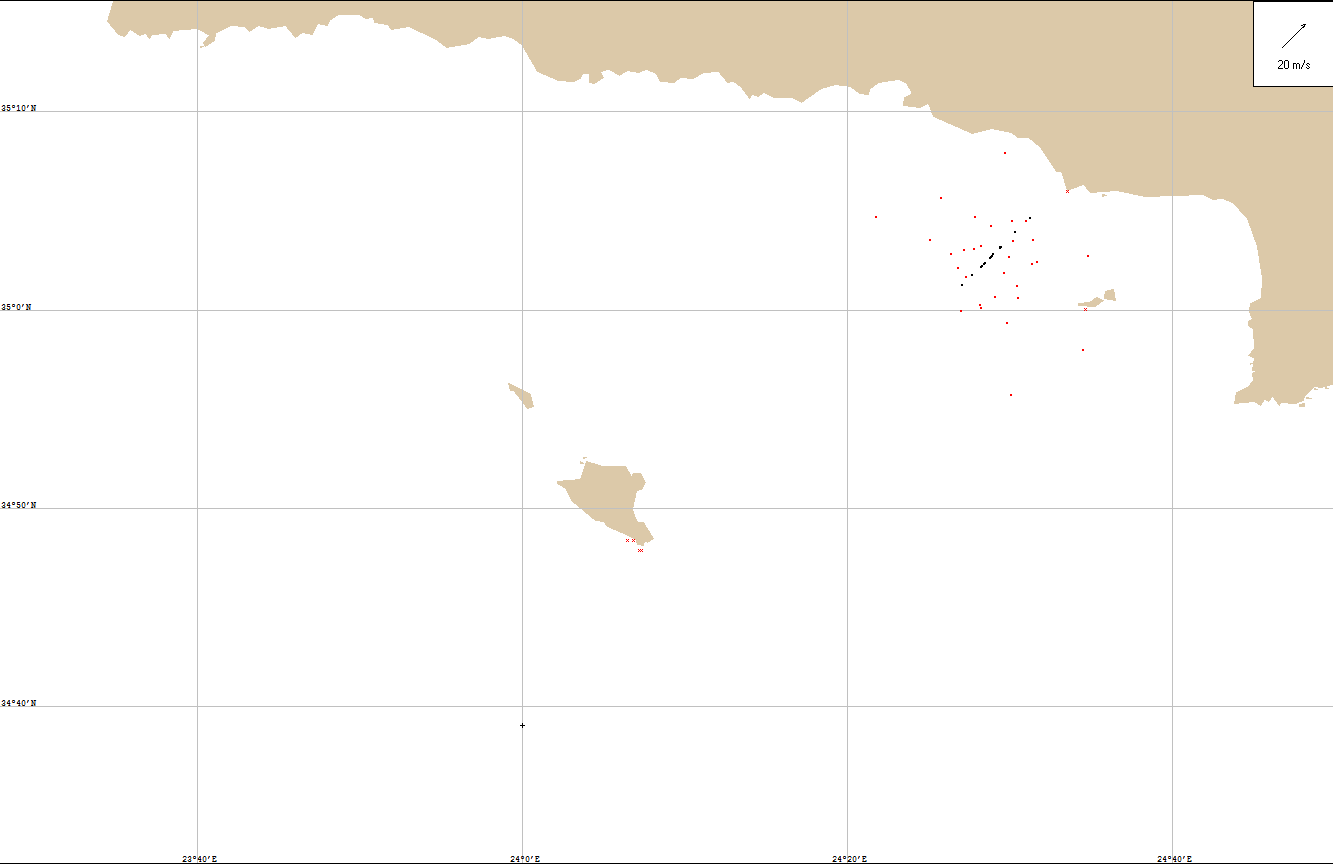
**d)**

Supplementary Figure 3 – Example of ADIOS models for a location south of Gavdos Island, offshore Greece (Scenario 6 in Supplementary Table 1). ADIOS is an oil weathering model produced by the National Oceanic and Atmospheric Administration (NOAA), i.e. an oil spill response tool that models how different types of oil weather (undergo physical and chemical changes) in the sea. Parameters considered in Scenario 6 included:

1. The oil remaining on the ship was 0% of the total released amount after 24 h.

2. Oil dispersion amounted to ~2.5% of the total oil released after 24 h.

3. Evaporation was able to dissipate ~ 98% of the total volume of oil after 24 h.

4. The Airborne Benzene Concentration was ~ 0.015ppm after 24 h.

5. The water content in oil was ~ 90% after 24 h. Emulsification is important.

6. Oil viscosity increased to ~ 50,500 cSt after 24 h.

7. Oil density was ~ 1020 kg/cu.m after 24 h.

8. There is strong evidence that the spill will arrive on the coast on 24/01/2018 03:03 UTC.


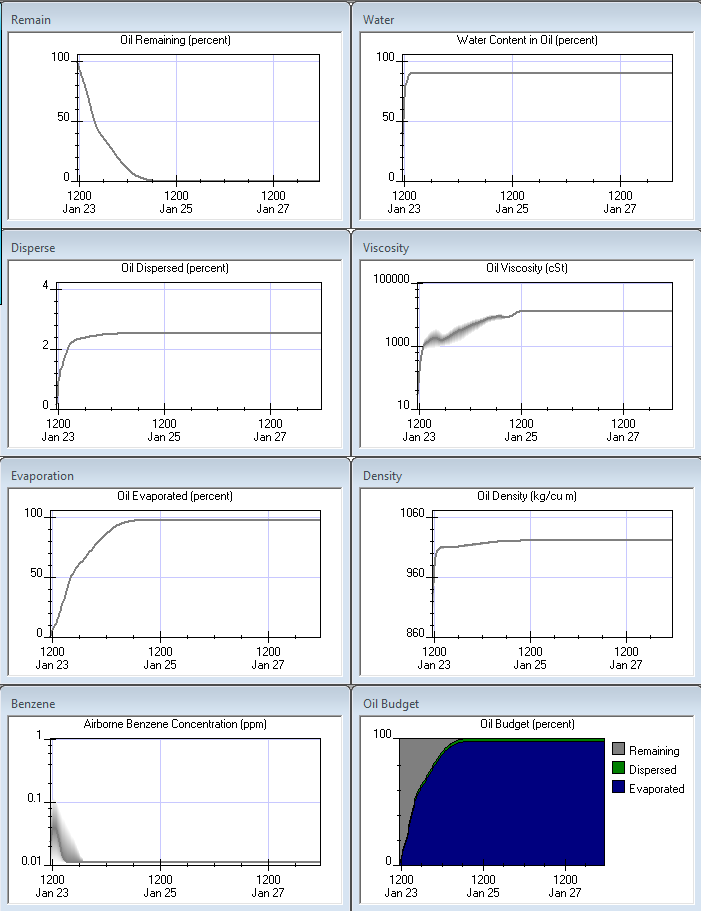

Supplement: Supplementary file 2 — Supplementary Figures. [file 41598_2021_82421_MOESM2_ESM.docx]
