# Supplementary material for: Scientific, societal and pedagogical approaches to tackle the impact of climate change on marine pollution
Source: Sci Rep. 2021 Feb 3;11:2927. doi: 10.1038/s41598-021-82421-y (PMC7858591; doi:10.1038/s41598-021-82421-y)
Supplement: Supplementary file 4 — Supplementary Movie Legend. [file 41598_2021_82421_MOESM4_ESM.docx]

Supplementary Movie 1 – Animated movie showing the results of the GNOME models for a location south of Gavdos Island, offshore Greece (Scenario 6 in Supplementary Table 1). See also Supplementary Figure 2, which highlights specific time steps in this simulation.
