# Supplementary material for: Scientific, societal and pedagogical approaches to tackle the impact of climate change on marine pollution
Source: Sci Rep. 2021 Feb 3;11:2927. doi: 10.1038/s41598-021-82421-y (PMC7858591; doi:10.1038/s41598-021-82421-y)
Supplement: Supplementary file 5 — Supplementary Table 1. [file 41598_2021_82421_MOESM5_ESM.docx]

**Scientific, societal and pedagogical approaches to tackle the impact of climate change on marine pollution**

Tiago M. Alves^1,*^, Eleni Kokinou^2,3^, Marie Ekström^1^, Andreas Nikolaidis^4^, Georgios C. Georgiou^4^, Anastasia Miliou^5^

1) 3D Seismic Lab – School of Earth and Ocean Sciences, Cardiff University – Main Building, Park Place, Cardiff, CF10 3AT, United Kingdom ([alvest@cardiff.ac.uk](mailto:alvest@cardiff.ac.uk))

2) Laboratory of Applied Geology and Hydrogeology, Department of Agriculture, Hellenic Mediterranean University, P.O. BOX 1939, 71004, Heraklion, Crete, Greece ([ekokinou@hmu.gr](mailto:ekokinou@hmu.gr))

3) Foundation for Research and Technology-Hellas, Institute of Computer Science, 70013 Heraklion, Crete, Greece

4) Oceanography Centre, University of Cyprus, P.O. Box 20537, 1678 Nicosia, Cyprus (and@ucy.ac.cy, georgios@ucy.ac.cy)

5) Archipelagos Institute of Marine Conservation, P.O. Box 42, Pythagorio 83 103, Samos, Greece

Supplementary Table 1 – Examples of pollution scenarios modeled in this work using GNOME and ADIOS. This list corresponds to the first-stage simulations completed to compile the e-game and teachers’ training curriculum. Together with these scenarios, four other accidents were considered for the Central North Sea and the Netherlands in a second stage, and later included in the Sea4All e-learning platform. One extra accident scenario was considered for the Gulf of Corinth, Greece. See the location of all pollution scenarios in the supplementary .KMZ file provided.

| **Title** |
| --- |
| Scenario 1 - Ship collision in Limassol |
| Scenario 2 - Ship collision in Egypt |
| Scenario 3 - Oil spill in Israel |
| Scenario 4 - Capsized ship in South Turkey |
| Scenario 5 – Oil spill in Rhodes |
| Scenario 6 - Storm in Gavdos |
| Scenario 7 - Plastics in Kithira |
| Scenario 8 - Fuel spill in Andros |
| Scenario 9- Fuel spill in the Central Aegean Sea |
| Scenario 10- Grounding in the Aegean Sea |
| Scenario 11- Drilling mud in Patras |
| Scenario 12- Plastic pollution in Romania |
| Scenario 15- Diesel spill in Georgia |
| Scenario 16- Ship collision off Turkey |
| Scenario 19- Oil Spill in the Celtic Sea |
| Scenario 20- Ship grounding on the Shetland Islands |
| Scenario 21- Oil spill in the Atlantic |
| Scenario 22- Power plant accident in Famagusta |
| Scenario 23- Sunken ship in Vasilikos |
| Scenario 24- Oil spill in Saronic Gulf |
| Scenario 25- Pipeline accident in SE Turkey |
| Scenario 27- Litter in Samos |
| Scenario 28- Litter in Lipsis |
| Scenario 29- Litter in Anidro |
| Scenario 30- Litter in Lesvos |
| Scenario 31- Litter in Crete |
| Scenario 32- Litter in Cyprus |
| Scenario 33- Oil spill in Agathonisi |
| Scenario 34- Oil spill in Santorini |
| Scenario 35- Oil spill in Mykonos |
| Scenario 36- Oil spill in Zakynthos |
| Scenario 37- Oil spill in Cape Greco |
| Scenario 38- Oil spill in Rimini |
| Scenario 39- Litter in Majorca |
| Scenario 40- Plastics in Santorini |
| Scenario 41- Litter in Kastellorizo |
| Scenario 43- Ghost nets in Thermaic Gulf |
| Scenario 44- Litter in Andros |
| Scenario 45- Urban litter in Athens |
| Scenario 46- Capsized ship in Felixstowe |

**Scenario 1 - Ship collision in Limassol**

**Description:**

Scenario based on a ship collision at Limassol, Cyprus (position LAT: 34^o^ 25N; LON: 33^o^ 38E) on 25 March 2018 at 02:00 UTC. The released cargo is cheifly composed of plastic material.

The cargo released into the sea was simulated as 200 metric tons of fuel oil (API: 12.3), spilled instantaneously. The simulation considered a period of 45 hours.

**Scenario 2 - Egypt ship collision**

**Description:**

Oil spill scenario near the Suez Canal, Egypt, associated with a ship collision (position LAT: 31° 26.44'N; LON: 32° 14.02'E) on 12 April 2018 at 10:00 UTC.

The oil released was simulated as 4,000 m^3^ of gasoline, spilled instantaneously. The simulation considered a period of 48 hours.

**Scenario 3 - Oil spill in Israel**

**Description:**

Scenario based on an oil spill occurring to the west of Haifa, Israel (position LAT: 32° 49.38'N; LON: 33° 36.05‘W) on 25 April 2018 at 05:00 UTC.

The oil released into the sea was simulated as 5,000 barrels of fuel oil (1 barrel = 159 liters) of heavy fuel oil #6 (API 12.3), spilled instantaneously. The simulation considered a period of 87 hours.

**Scenario 4 - Capsized ship in South Turkey**

**Description:**

Storm capsizing a ship south of Antalya, Turkey, with the appearance of an oil spill (position LAT: 35° 14.72'N; LON: 29° 28.53'E) on 06 May 2018 at 22:00 UTC.

The oil released into the sea was simulated as 43,000 m^3^ of gasoline oil (API 32.5), spilled instantaneously. The simulation considered a period of 61 hours (~2.5 days).

**Scenario 5 – Oil spill in Rhodes**

**Description:**

Oil spill southeast of Rhodes (position LAT: 35° 53.65'N; LON: 28° 35.63'E) on 08 May 2018 at 12:00 UTC.

The oil released into the sea was simulated as 4,000 m^3^ of heavy fuel oil #6 (API 12.3), spilled instantaneously. The simulation considered a period of 114 hours (~ 5 days).

**Scenario 6 - Storm in Gavdos**

**Description:**

Oil spill offshore Gavdos, Greece, after a storm led a ship into trouble (position LAT: 34° 31.12'N; LON: 23° 46.96'E) on 09 May 2018 at 01:00 UTC.

The oil released into the sea was simulated as 29,000 metric tons of diesel oil (API 34.5), spilled instantaneously. The simulation considered a period of 96 hours.

**Scenario 7 - Plastics in Kithira**

**Description:**

Ship collision off Kithira, Greece (position LAT: 35° 40.32'N; LON: 23° 14.23'E) on 13 May 2018 at 11:00 UTC. The sea cargo was composed of plastic materials.

The cargo released was simulated as 35,000 metric tons of fuel oil (API 32.5), spilled instantaneously. The simulation considered a period of 26 hours.

**Scenario 8 - Fuel spill in Andros**

**Description:**

Oil spill near Andros, Greece, due to a cargo ship becoming grounded on an islet (position LAT: 37° 42.44'N; LON: 24° 41.30'E) on 13 May 2018 at 11:00 UTC.

The oil released into the sea was simulated as 10,000 metric tons of Gasoline oil (API: 32.5), spilled instantaneously. The simulation considered a period of 48 hours.

**Scenario 9 - Fuel spill in the Central Aegean Sea**

**Description:**

Oil spill in the Central Aegean Sea, between Lesvos and the island of Agios Efstratios, Greece, due to a gas blowout (position LAT: 39° 9.98'N; LON: 25° 6.38'E) on 15 May 2018 at 12:00 UTC.

The oil released into the sea was simulated as 40,000 metric tons of Gasoline oil (API: 32.5), spilled instantaneously. The simulation considered a period of 48 hours.

**Scenario 10 - Grounding in the Aegean Sea**

**Description:**

Floating objects released in the Central Aegean, Greece, due to a grounded ship (position LAT: 39° 42.51'N; LON: 24° 23.68'E) on the 17 May 2018 at 01:00 UTC.

The objects released into the sea were simulated as 30,000 metric tons of fuel oil #6 (API 12.3), spilled instantaneously. The simulation considered a period of 28 hours.

**Scenario 11- Drilling mud in Patras**

**Description:**

Mud and oil released near Patras, Greece, due to the loss of drilling mud during the drilling operations (position LAT: 38° 1.26'N; LON: 20° 59.10'E) on the 20 May 2018 at 10:00 UTC.

The mud and oil released close to the drilling rig were simulated as 2,000 metric tons of fuel oil #6 (API 12.3), spilled instantaneously. The simulation considered a period of 5 days.

**Scenario 12- Plastic pollution in Romania**

**Description:**

Accumulation of plastic litter occurred on the sea floor of Constanţa harbor, Romania, at the start of the tourist season (position LAT: 44° 13.63'N; LON: 28° 48.52'E) on 21 May 2018 at 00:00 UTC.

The plastic pollution was simulated as 6,350 metric tons of diesel oil (API: 32.5), spilled instantaneously. The simulation considered a period of 48 hours.

**Scenario 15- Diesel spill in Georgia**

**Description:**

Oil spill scenario in the Black Sea due to the leakage of oil near a harbor (position LAT: 41° 29.12'N; LON: 41° 9.25'E) on 28 May 2018 at 22:00 UTC

The oil released into the sea was simulated as 60,000 metric tons of diesel oil (API: 33.5), spilled instantaneously. The simulation considered a period of 64 hours.

**Scenario 16- Ship collision off Turkey**

**Description:**

Oil spill occurring near the Bosphorus Strait, Turkey, due to a collision between two oil tankers (position 41° 13.09'N; LON: 29° 40.84'E) on 20 May 2018 at 22:00 UTC.

The oil released into the sea was simulated as 59,000 metric tons of heavy fuel oil #6 (API: 12.3), spilled continuously for the first 48 hours. The simulation considered a period of 96 hours.

**Scenario 19- Oil Spill in the Celtic Sea**

**Description:**

Oil spill scenario based on an accident with a cruise ship near Cornwall, UK (position LAT: 49° 47.01'N; LON: 7° 9.02'W) on 01 June 2018 at 12:00 UTC.

The oil released into the sea was simulated as 60,000 metric tons of diesel oil (API: 33.5), spilled instantaneously. The simulation considered a period of 96 hours.

**Scenario 20- Ship grounding on the Shetland Islands**

**Description:**

Oil spill occurred in Shetland, UK, due to the grounding of the M/V Braer oil tanker (LAT: 59° 43.83'N; LON: 1° 23.80'W) on 03 June 2018 at 12:00 UTC, i.e. under distinct conditions from the original accident in 1993.

The oil released into the sea was simulated as 70,000 metric tons of diesel oil (API: 33.5), spilled instantaneously. The simulation considered a period of 140 hours.

**Scenario 21- Oil spill in the Atlantic**

**Description:**

Oil spill scenario in Atlantic waters based on the “Prestige” accident (position LAT: 42° 42.85'N; LON: 12° 48.78‘W) on 17 April 2018 at 12:00 UTC, under distinct conditions from the original accident in 2004.

The oil released into the sea was simulated as 77,000 m^3^ of fuel oil #6 (API: 12.3), spilled instantaneously. The simulation considered a period of 10 days.

**Scenario 22- Power plant accident in Famagusta**

**Description:**

Oil spill near a power plant close to Famagusta, Cyprus (position: LAT: 35° 18.61'N; LON: 33° 59.50'E) on 09 May 2018 at 5:00 UTC.

The oil released into the sea was simulated as 5,000 metric tons of heavy fuel oil #6 (API 12.3), spilled instantaneously. The simulation considered a period of 34 hours.

**Scenario 23- Sunken ship in Vasilikos**

**Description:**

Oil spill in the area south of Vasilikos, Cyprus, due to a ship accident (position LAT: 34° 20.8'N; LON: 28° 33.27.1'E) on 09 May 2018 at 14:00 UTC.

The oil released into the sea was simulated as 100 metric tons of fuel oil #6 (API 12.3), spilled instantaneously. The simulation considered a period of 7 days.

**Scenario 24- Oil spill in Saronic Gulf**

**Description:**

Ship accident with an ensuing oil spill in the Saronic Gulf, near Athens, Greece (position LAT: 37° 55.90'N; LON: 23° 34.02'E) on 01 June 2018 at 03:00 UTC.

The oil released into the sea was simulated as 170 metric tons of diesel oil (API: 33.5), spilled instantaneously. The simulation considered a period of 45 hours.

**Scenario 25- Pipeline accident in SE Turkey**

**Description:**

Rupture of a new pipeline in Adana, Turkey (position LAT: 36° 47.33'N; LON: 35°57.91'E) on 01 June 2018 at 13:00 UTC, with subsequent spill of oil.

The oil released into the sea was simulated as metric tons of fuel oil #6 (API: 12.3), continuously spilled for the first 9 hours. The simulation considered a period of 52 hours.

**Scenario 27- Litter in Samos**

**Description:**

Marine litter accumulated near Samos, Greece (position LAT: 37° 42.05'N; LON: 26° 58.56'E) on 04 June 2018 at 15:00 UTC.

The litter released onto the sea was simulated as 10,000 metric tons of gasoline oil (API: 32.5), spilled instantaneously. The simulation considered a period of 12 hours.

**Scenario 28- Litter in Lipsis**

**Description:**

Marine litter accumulated near Lipsis, Greece (position LAT: 37° 19.00'N; LON: 26° 42.32'E) on 06 June 2018 at 18:00 UTC.

The litter released onto the sea was simulated as 4,000 metric tons of gasoline oil (API: 32.5), spilled instantaneously. The simulation considered a period of 48 hours.

**Scenario 29- Litter in Anidro**

**Description:**

Accumulated litter offshore Anidro, Greece (position LAT: 37° 19.96'N; LON: 26° 29.99'E) on 07 June 2018 at 10:00 UTC.

The litter released was simulated as 2,500 metric tons of gasoline oil (API: 32.5), spilled instantaneously. The simulation considered a period of 31 hours.

**Scenario 30- Litter in Lesvos**

**Description:**

Scenario based on the transport of dropped plastic bottles from the mountain into the sea of Lesvos Island, Greece (position: LAT: 39° 6.86'N; LON: 25° 54.84'E) on 08 June 2018 at 00:00 UTC. With the effect of sunlight and wave action, the plastic rapidly breaks down into small pieces and enters the various levels of the marine food chain.

The plastic reaching the sea was simulated as 2,500 metric tons of gasoline oil (API: 32.5), spilled instantaneously. The simulation considered a period of 17 hours.

**Scenario 31- Litter in Crete**

**Description:**

Scenario based on the transport of dropped plastic bottles from the mountains into the sea of Crete, Greece (position: LAT: 35° 21.12'N; LON: 25° 15.74'E) on 12 June 2018 at 22:00 UTC. With the effect of sunlight and wave action, the plastic rapidly breaks down into small pieces and enters various levels of the marine food chain.

The plastic reaching the sea was simulated as 2,500 metric tons of gasoline oil (API: 32.5), spilled instantaneously. The simulation considered a period of 20 hours.

**Scenario 32- Litter in Cyprus**

**Description:**

Scenario based on plastic bottles transported from the coast to the sea of Cyprus (position: LAT: 35° 12.73'N; LON: 32° 48.68'E) on 12 June 2018 at 18:00 UTC.

The plastic bottles reaching the sea simulated as 2,500 metric tons of gasoline oil (API: 32.5), spilled instantaneously. The simulation considered a period of 30 hours.

**Scenario 33- Oil spill in Agathonisi**

**Description:**

Oil spill offshore Agathonisi island, Greece, due to a capsized ship (position LAT: 37° 25.24'N; LON: 26° 54.99'E) on 14 June 2018, at 12:00 UTC.

The oil released into the sea simulated as 450 metric of fuel oil #6 (API 12.3), continuously spilled for the first 2 hours. The simulation considered a period of 153 hours.

**Scenario 34- Oil spill in Santorini**

**Description:**

Oil spill offshore Santorini island, Greece, due to a ship collision and subsequent sinking (position LAT: 36° 24.61'N; LON: 25° 13.94'E) on 16 June 2018 at 13:00 UTC.

The oil released into the sea simulated as 450 metric tons of fuel oil #6 (API: 12.3), continuously spilled for the first 2 hours. The simulation considered a period of 44 hours.

**Scenario 35- Oil spill in Mykonos**

**Description:**

Oil spill near Mykonos island, Greece, due to the sinking of a cargo ship (position LAT: 37° 28.77'N; LON: 25° 30.72'E) on the 20 June 2018 at 09:00 UTC.

The oil released into the sea simulated as 450 metric tons of fuel oil #6 (API: 12.3), spilled instantaneously. The simulation considered a period of 31 hours.

**Scenario 36- Oil spill in Zakynthos**

**Description:**

Oil spill near Zakynthos island, Greece, due to a vessel leakage (position LAT: 37° 57.2'N; LON: 20° 34.39'E ) on 21 June 2018 at 22:00 UTC.

The oil released into the sea simulated as 1500 metric tons of fuel oil #6 (API: 12.3), spilled instantaneously. The simulation considered a period of 36 hours.

**Scenario 37- Oil spill in Cape Greco**

**Description:**

Leakage from recreation vessel developing an oil spill in Cape Greco, Cyprus (position LAT: 34° 53.68'N; LON: 34° 0.84'E) on 15 June 2018 at 18:00 UTC.

The oil released into the sea simulated as 300 metric tons of fuel oil #6 (API: 12.3), spilled instantaneously. The simulation for a period of 44 hours.

**Scenario 38- Oil spill in Rimini**

**Description:**

Oil spill near the beaches of Rimini, Italy, due to an illegal leakage from a sailing vessel (position LAT: 44° 5.9'N; LON: 12° 37.71'E) on 22 June 2018 at 10:00 UTC.

The oil released into the sea simulated as 1500 metric tons of fuel oil #6 (API: 12.3), spilled instantaneously. The simulation considered a period of 96 hours.

**Scenario 39- Litter in Majorca**

**Description:**

Plastic and debris released to sea during a cultural event near Palma de Mallorca, Spain (position LAT: 39° 31.7'N; LON: 2° 40.66'E) on 23 June 2018 at 00:00 UTC.

The plastic released in the sea was simulated as 1500 metric tons of fuel oil #6 (API: 12.3), spilled instantaneously. The simulation considered a period of 92 hours.

**Scenario 40- Plastics in Santorini**

**Description:**

Scenario based on plastic packaging and debris released next to Santorini island, Greece (position LAT: 36° 22.63'N; LON: 25° 23.45'E) on 17 June 2018 at 15:00 UTC.

The pollution event was simulated as 1,500 metric tons of gasoline oil (API: 32.5), spilled instantaneously. The simulation ran for a period of 44 hours.

**Scenario 41- Litter in Kastellorizo**

**Description:**

Scenario based on floating plastic debris dropped between Rhodos and Kastelorizo, Greece (position: LAT: 36° 20.02'N; LON: 28° 57.65'E) on 13 June 2018 at 10:00 UTC.

The plastic released in the sea was simulated as 1,500 metric tons of fuel oil #6 (API: 12.3), spilled instantaneously. The simulation considered a period of 182 hours.

**Scenario 43- Ghost nets in Thermaic Gulf**

**Description:**

After a marine accident, large drifting fishing nets appear near Thessaloniki, Thermaic Gulf, Greece (position 37° 48.74'N; LON: 24° 58.95'E) on 27 June 2018 at 14:00 UTC.

The drifting nets were simulated as 1,500 metric tons of gasoline oil (API: 32.5), spilled instantaneously. The simulation considered a period of 96 hours.

**Scenario 44- Litter in Andros**

**Description:**

Scenario based on the dropping of garbage pits into the sea next to Andros, Greece (position: LAT: 37° 48.74'N; LON: 24° 58.95'E) on 20 May 2018 at 19:00 UTC.

The garbage released in the sea was simulated as 1,500 metric tons of gasoline oil (API: 32.5), spilled instantaneously. The simulation considered a period of 29 hours.

**Scenario 45- Urban litter in Athens**

**Description:**

Scenario based on the accumulation of litter and plastic on an Athens beach after a popular music concert (position LAT: 37° 56.27'N; LON: 23° 40.75'E) on 13 May 2018 at 00:00 UTC.

The urban litter was simulated as 1,500 metric tons of gasoline oil (API: 32.5), spilled instantaneously. The simulation considered a period of 36 hours.

**Scenario 46- Capsized ship in Felixstowe**

**Description:**

Scenario based on floating debris released in the Felixstowe Harbour, UK, due to a capsized ship (position LAT: 49° 47.01'N; LON: 7° 9.02'W) on 01 June 2018 at 12:00 UTC.

The debris were simulated as 60,000 metric tons of diesel oil (API: 33.5), spilled instantaneously. The simulation considered a period of 96 hours.
