# Supplementary material for: Scientific, societal and pedagogical approaches to tackle the impact of climate change on marine pollution
Source: Sci Rep. 2021 Feb 3;11:2927. doi: 10.1038/s41598-021-82421-y (PMC7858591; doi:10.1038/s41598-021-82421-y)
Supplement: Supplementary file 6 — Supplementary Table 2. [file 41598_2021_82421_MOESM6_ESM.docx]

**Scientific, societal and pedagogical approaches to tackle the impact of climate change on marine pollution**

Tiago M. Alves^1,*^, Eleni Kokinou^2,3^, Marie Ekström^1^, Andreas Nikolaidis^4^, Georgios C. Georgiou^4^, Anastasia Miliou^5^

1) 3D Seismic Lab – School of Earth and Ocean Sciences, Cardiff University – Main Building, Park Place, Cardiff, CF10 3AT, United Kingdom ([alvest@cardiff.ac.uk](mailto:alvest@cardiff.ac.uk))

2) Laboratory of Applied Geology and Hydrogeology, Department of Agriculture, Hellenic Mediterranean University, P.O. Box 1939, 71004, Heraklion, Crete, Greece ([ekokinou@hmu.gr](mailto:ekokinou@hmu.gr))

3) Foundation for Research and Technology-Hellas, Institute of Computer Science, 70013 Heraklion, Crete, Greece

4) Oceanography Centre, University of Cyprus, P.O. Box 20537, 1678 Nicosia, Cyprus (and@ucy.ac.cy, georgios@ucy.ac.cy)

5) Archipelagos Institute of Marine Conservation, P.O. Box 42, Pythagorio 83 103, Samos, Greece

Supplementary Table 2 - Climate models underpinning future change signal in flood recurrence for selected catchments in the SWICCA dataset.

| **GCM** | **RCM** | **Institute** |
| --- | --- | --- |
| EC-Earth | RAC4 | SMHI |
| EC-Earth | RACMO22E | KNMI |
| HadGEM2-ES | RAC4 | SMHI |
| MPI-ESM-LR | REMO2009 | CSC |
